# Early disruption of nerve mitochondrial and myelin lipid homeostasis in obesity-induced diabetes

**DOI:** 10.1172/jci.insight.137286

**Published:** 2020-11-05

**Authors:** Juan P. Palavicini, Juan Chen, Chunyan Wang, Jianing Wang, Chao Qin, Eric Baeuerle, Xinming Wang, Jung A. Woo, David E. Kang, Nicolas Musi, Jeffrey L. Dupree, Xianlin Han

**Affiliations:** 1 Barshop Institute for Longevity and Aging Studies and; 2 Division of Diabetes, Department of Medicine, University of Texas Health Science Center at San Antonio, San Antonio, Texas, USA.; 3 School of Basic Medical Sciences, Zhejiang Chinese Medical University, Hangzhou, China.; 4 Byrd Alzheimer’s Center and Research Institute, USF Health Morsani College of Medicine, University of South Florida, Tampa, Florida, USA.; 5 Department of Anatomy and Neurobiology, School of Medicine, Virginia Commonwealth University, Richmond, Virginia, USA.; 6 Research Service, Hunter Holmes McGuire Veterans Affairs Medical Center, Richmond, Virginia, USA.

**Keywords:** Metabolism, Neuroscience, Demyelinating disorders, Diabetes, Neurological disorders

## Abstract

Diabetic neuropathy is a major complication of diabetes. Current treatment options alleviate pain but do not stop the progression of the disease. At present, there are no approved disease-modifying therapies. Thus, developing more effective therapies remains a major unmet medical need. Seeking to better understand the molecular mechanisms driving peripheral neuropathy, as well as other neurological complications associated with diabetes, we performed spatiotemporal lipidomics, biochemical, ultrastructural, and physiological studies on PNS and CNS tissue from multiple diabetic preclinical models. We unraveled potentially novel molecular fingerprints underlying nerve damage in obesity-induced diabetes, including an early loss of nerve mitochondrial (cardiolipin) and myelin signature (galactosylceramide, sulfatide, and plasmalogen phosphatidylethanolamine) lipids that preceded mitochondrial, myelin, and axonal structural/functional defects; started in the PNS; and progressed to the CNS at advanced diabetic stages. Mechanistically, we provided substantial evidence indicating that these nerve mitochondrial/myelin lipid abnormalities are (surprisingly) not driven by hyperglycemia, dysinsulinemia, or insulin resistance, but rather associate with obesity/hyperlipidemia. Importantly, our findings have major clinical implications as they open the door to novel lipid-based biomarkers to diagnose and distinguish different subtypes of diabetic neuropathy (obese vs. nonobese diabetics), as well as to lipid-lowering therapeutic strategies for treatment of obesity/diabetes-associated neurological complications and for glycemic control.

## Abstract 2

Spatiotemporal lipidomics, biochemical, ultrastructural, and physiological characterization of peripheral and central nervous system tissue from multiple diabetic pre-clinical models.

## Introduction

Diabetic peripheral neuropathy, the most common complication of diabetes mellitus, is a neurodegenerative disease of the PNS that represents a significant source of morbidity and mortality associated with diabetes (1, 2). The signs and symptoms of diabetic neuropathy vary, depending on the type of neuropathy and which nerves are affected (peripheral nerves, autonomic nerves, and/or cranial nerves), ranging from pain and numbness in the extremities to major organ dysfunction. At present, there are no approved disease-modifying therapies for diabetic neuropathy. Current treatment options alleviate pain but do not stop disease progression. In addition, diabetes has also been associated with disturbances in the CNS, including cerebrovascular lesions and cognitive dysfunction (3–6). In fact, diabetes is a well-established risk factor for vascular dementia and Alzheimer’s disease (7–11).

Although hyperglycemia has been historically considered the driving force underlying the development of diabetic neuropathy, cumulative evidence during the last decade has contended against this assumption by implicating additional components of metabolic syndrome (e.g., obesity, hyperlipidemia, and insulin resistance) with the etiology of diabetic neuropathy (12–17). Thus, better understanding the molecular mechanisms underlying metabolic syndrome–associated peripheral neuropathy, as well as developing novel and more effective therapies, are of urgent public health importance.

Loss of myelinated nerve fibers has been repeatedly documented in diabetic neuropathy (18, 19). Demyelination has long been described as a pathological feature of clinical and long-term experimental diabetic neuropathy (19–21). Despite controversy as to whether demyelination or axonal loss is the primary lesion in diabetic neuropathy, the integral role played by Schwann cells in the maintenance of peripheral nerve structure/function shows that these myelinating cells play a central role during disease pathogenesis (19). Mitochondrial dysfunction has also been implicated as an etiological factor in diabetic neuropathy (22–25). The fact that several peripheral neuropathies are caused by hereditary mutations of genes that are directly involved in mitochondrial dynamics directly links mitochondrial dysfunction to peripheral nerve pathology (26).

Considering that lipids not only are critical components of membrane-rich mitochondrial and myelin structures, but also play essential functional roles in both of these cellular compartments, we undertook a lipidomics approach to unravel the effects of diabetes on nerve lipid metabolism. Assessment of diet-induced, genetically induced, and drug-induced diabetic animal models revealed a potentially novel molecular fingerprint associated with obesity-induced diabetic neuropathy: an early disruption of nerve mitochondrial and myelin lipid homeostasis that, surprisingly, is not driven by hyperglycemia, dysinsulinemia, or insulin resistance. We provide strong evidence that in addition to hyperglycemia and insulin resistance, other components of metabolic syndrome, particularly obesity and/or hyperlipidemia, can drive nerve damage and contribute to the development of peripheral neuropathy and other neurological complications. Our studies unraveled a potentially novel subtype of peripheral neuropathy associated with obesity-induced diabetes characterized by an early disruption of mitochondrial and myelin lipid homeostasis. These findings have important clinical implications because they suggest that different subtypes of diabetic neuropathy (obese versus nonobese diabetics) display different etiologies and, thus, could be diagnosed and treated differently.

## Results

### Early and progressive losses of myelin lipids in db/db mice, from the periphery to the brain.

Galactosylceramide (cerebroside) and its sulfated form, sulfatide, are highly enriched in myelin and represent bona fide myelin signature lipids. In fact, the concentration of cerebroside in the brain has been shown to be directly proportional to the amount of myelin present (27). Another peculiarity in the lipid composition of myelin has to do with the composition of phosphatidylethanolamines (PEs). Whereas in most membranes PE is primarily present in its diacyl form, myelin is characterized by a high content of plasmalogen PE containing dominant oleic acid (18:1) (28, 29). With this in mind, we performed spatiotemporal multidimensional mass spectrometry–based (MDMS) shotgun lipidomics analyses from PNS (dorsal root ganglia and/or sciatic nerves) and CNS (spinal cord and brainstem) tissue homogenates of WT mice and mice homozygous for the diabetes spontaneous mutation *Lepr^db^* (*db/db*) at different time points (1, 2, and 4 months of age).

**Figure 1:**
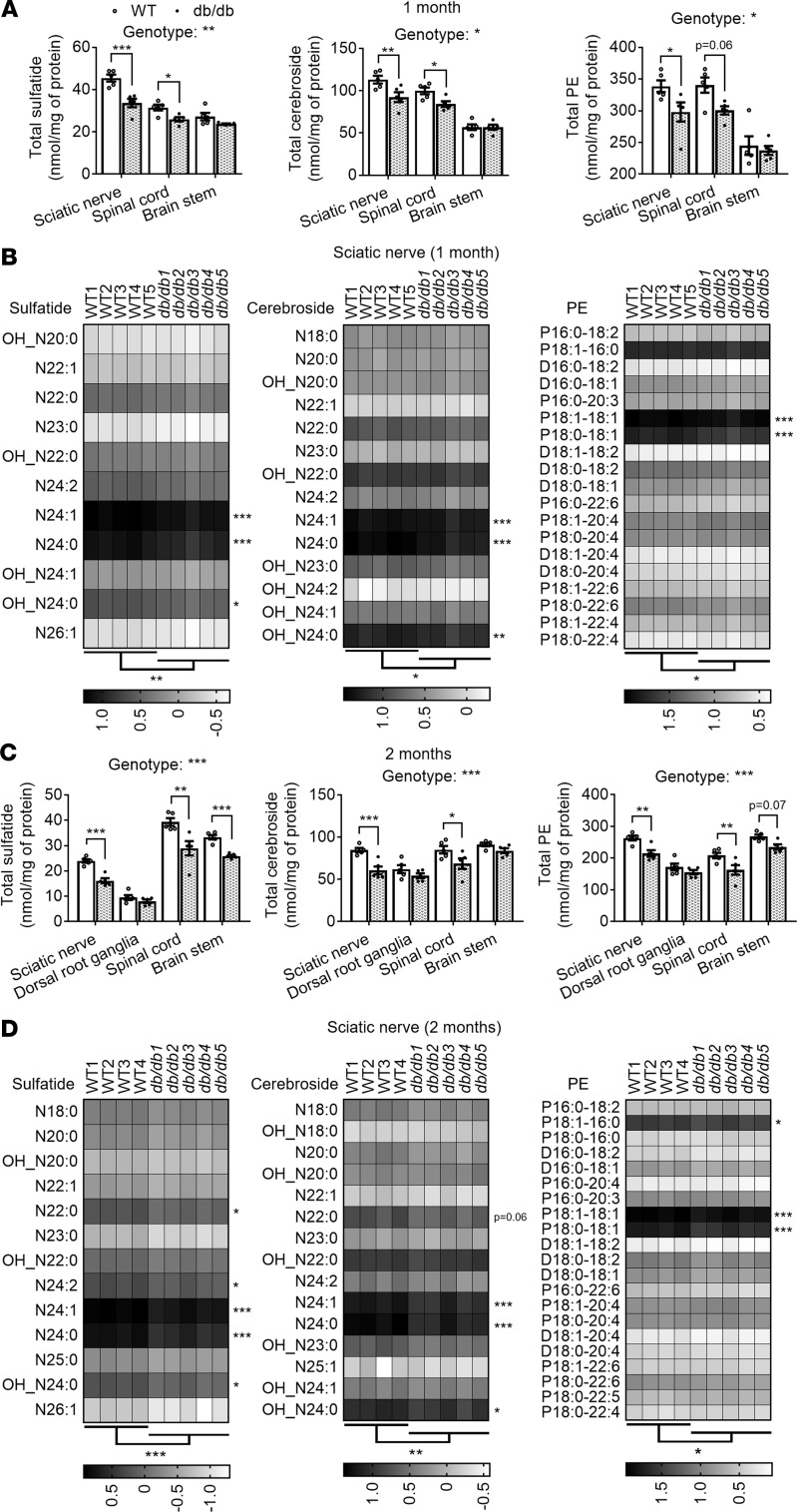
Dying-back depletion of myelin-enriched lipids in obese diabetic mice. Sciatic nerve, dorsal root ganglia, spinal cord, and brainstem tissue from WT and *db/db* male mice were dissected, flash-frozen, homogenized, and lipid extracted. Sulfatide, cerebroside, and phosphatidylethanolamine (PE) levels were assessed by multidimensional mass spectrometry–based shotgun lipidomics for 1- (**A** and **B**) and 2-month-old (**C** and **D**) mice. Total levels of each lipid class are displayed as dot plots (WT: open circles/bars; *db/db*: filled circles/bars); data represent the mean ± SEM of *n* = 4–5 mice/genotype (**A** and **C**). Total levels of each lipid class were compared between genotypes for every tissue examined at each time point using 2-way ANOVA (genotype effect *P* values specified on top of each graph) and Holm-Šidák multiple-comparisons tests (adjusted *P* values specified on top of brackets). Molecular species masses expressed as nmol/mg of protein were log transformed and displayed as gray scale heatmaps (**B** and **D**). Low-abundance lipid species that made ≤1% of the total class content are not shown and were not included in statistical analyses. Although a specific mass peak may represent >1 cardiolipin molecular species, for simplicity heatmaps display only 1 (most common) lipid species for each mass (row). Molecular species within each lipid class were compared using 2-way ANOVA and Holm-Šidák multiple-comparisons tests on GraphPad Prism 7. Statistics displayed below the heatmaps comparing genotypes and next to the molecular species are based on nontransformed data. N, amide-linked; P, plasmalogen or alkenyl-acyl-linked; D, diacyl-linked. *P < 0.05, **P < 0.01, ***P < 0.001.

At the earliest time point analyzed (1 month), *db/db* mice were already identifiably obese (+33% increase in body weight) and hyperinsulinemic (+7.5-fold increase in nonfasted serum insulin) but were still normoglycemic (normal nonfasted blood glucose) (Supplemental Figure 1; supplemental material available online with this article; https://doi.org/10.1172/jci.insight.137286DS1). Remarkably, lipidomics results revealed a very early loss of myelin signature lipids in *db/db* nervous tissue compared with WT controls at this prediabetic obese stage. Specifically, total sulfatide and cerebroside levels (myelin signature lipid classes) were significantly decreased (–25% and –18%, respectively) in *db/db* sciatic nerve tissue (Figure 1A). In addition, the 2 major plasmalogen (P) PE species (P18:0-18:1 and P18:1-18:1) were significantly reduced (–27% and –12%, respectively) (Figure 1B). These 2 major plasmalogen PE species make up about half the total mass of PE in sciatic nerve tissue under physiological conditions. Consequently, total PE content was also significantly decreased (–11%) in diabetic mice (Figure 1A).

Surprisingly, significant decreases were also observed early on in spinal cord tissue of *db/db* mice. Again, a global but less intense loss of myelin lipids was observed; total sulfatide and cerebroside levels were significantly decreased (–18% and –16%, respectively), while total PE levels tended to decrease (–12%) (Figure 1A). As in sciatic nerves, major species of these myelin-enriched lipid classes were significantly reduced (*P* < 0.05, data not shown). No significant changes in myelin signature lipids were observed in the brain (brainstem) at this early stage (Figure 1A).

Next, we assessed whether this early global loss of myelin lipids in diabetic mice worsened with age/disease progression. At 2 months of age, *db/db* mice were dramatically obese (+66% in body weight) and displayed severe hyperinsulinemia (+6-fold in nonfasted blood insulin) and hyperglycemia (+2.6-fold in nonfasted blood glucose), indicative of severe insulin resistance (Supplemental Figure 1). Importantly, at this stage, the reduction of myelin-enriched lipids in *db/db* sciatic nerve tissue was exacerbated. Specifically, total sulfatide, cerebroside, and PE levels were further reduced (–33%, –29%, –19%, respectively), with all major molecular species being significantly reduced (Figure 1, C and D).

To gain further insights into the effects of diabetes on the PNS lipidome, at this time point we also isolated dorsal root ganglia (DRG), where neuronal cell bodies are clustered. Although total sulfatide, cerebroside, or PE levels were not significantly reduced in DRG (Figure 1C), statistical analysis of their molecular species revealed a significant genotype effect for each of these myelin-enriched lipid classes, with at least 1 of their major molecular species being significantly decreased in diabetic mice (Supplemental Figure 2).

Myelin lipid losses were also aggravated in the CNS as diabetes progressed. In the spinal cord of 2-month-old *db/db* mice, total sulfatide and cerebroside levels were further reduced (–26% and –19%, respectively), as well as major spinal cord P PE species (P18:1-16:0, P18:1-18:1, P18:0-18:1), which led to a significant decrease of total PE content (–22%) (Figure 1, C and D). At this stage, even the brain started to display significant deficiencies of myelin-enriched lipids, with total sulfatide levels being significantly reduced in the brainstem (–22%) and total PE levels trending to decline (–12%) (Figure 1C).

The magnitude of this global loss of myelin lipids eventually plateaued with disease progression, as the reductions in total sulfatide, cerebroside, and major P PE species in the sciatic nerves of 4-month-old *db/db* mice were similar to or slightly milder than those observed at 2 months of age (Supplemental Figure 3). At 4 months of age, *db/db* mice continued displaying dramatic obesity (+60% in body weight) and hyperinsulinemia (+5.9-fold in nonfasted blood insulin); however, at this stage, hyperglycemia was milder compared with its peak at 2 months of age (+1.9-fold in nonfasted blood glucose) (Supplemental Figure 1). These weight/insulin/glucose results are consistent with the phenotypes described for B6-db mice (*db/db* mice in C57BL/6J background) by The Jackson Laboratory and the literature (30, 31).

On the other hand, total levels of phosphatidylcholine (PC), a major cellular phospholipid, were not significantly altered in PNS or CNS tissue at any of the time points analyzed (Supplemental Figure 4). Thus, the described loss of myelin lipids in obese-associated diabetes does not seem to be driven by an overall reduction of nerve lipid biosynthesis. Supporting this notion, RNA expression studies revealed that lipid biosynthesis-related genes (i.e., *Srebp1*, *Srebp2*, and several of their target genes) were not reduced in 2-month-old diabetic mice (Supplemental Figure 5).

**Figure 2:**
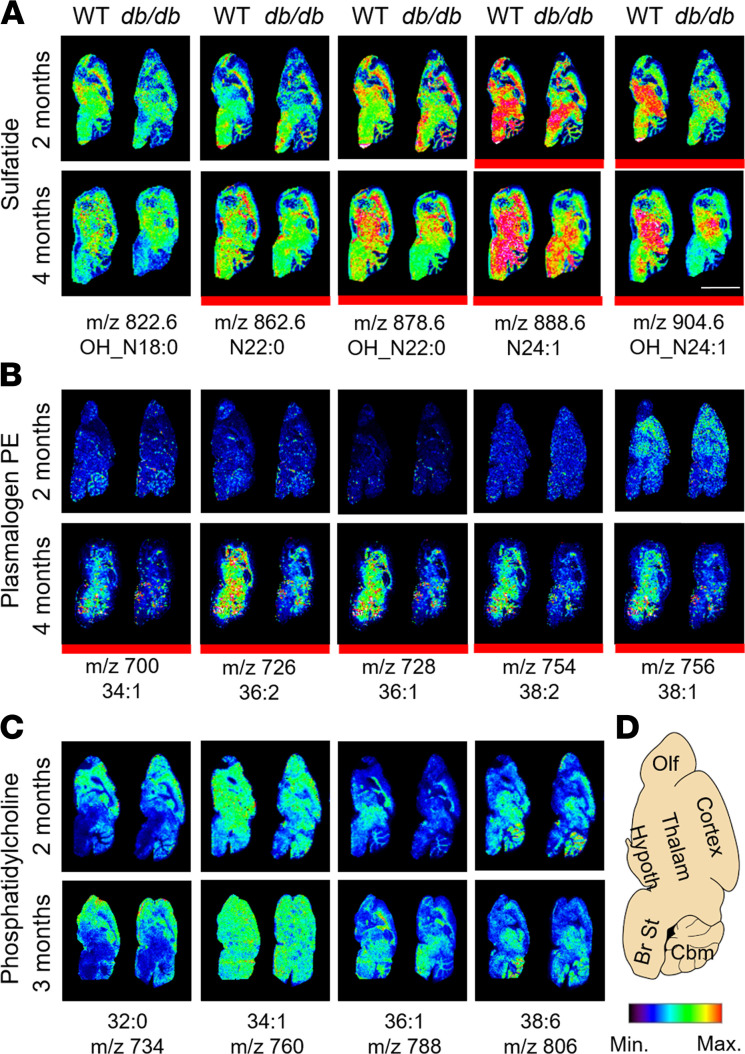
Depletion of myelin-enriched lipids in the brains of obese diabetic mice. Whole brains from 2- to 4-month-old WT and *db/db* male mice were dissected, frozen, and sectioned sagittally (10 μm). Representative MALDI mass spectrometry imaging (MSI) maps of major sulfatide (**A**), P PE (**B**), and phosphatidylcholine (PC) (**C**) molecular species from *n* = 2–3 male mice/genotype/time point, except for 3/4-month plasmalogen PE and PC, where only 1 mouse per genotype was assessed. Lipid molecular species that displayed evident and consistent genotype differences are highlighted by a red thick line below the image. MALDI MSI resolution: 150 μm; scale bar (white): 5 mm. (**D**) Mouse brain schematic. Olf, olfactory lobe; Thalam, thalamus; Hypoth, hypothalamus; Br St, brainstem; Cbm, cerebellum.

Furthermore, we performed matrix-assisted laser desorption/ionization (MALDI) mass spectrometry imaging studies on WT and *db/db* brains at 2–4 months of age (Figure 2). Consistent with our MDMS shotgun lipidomics data, we observed a decrease of highly abundant sulfatide species (N24:1 and OH N24:1) in the brains of 2-month-old *db/db* mice compared with controls, particularly within the brainstem (Figure 2A). At 4 months of age, clear qualitative decreases of all major sulfatide and plasmalogen PE species were consistently observed throughout the whole brains of *db/db* mice (Figure 2, A and B). On the other hand, other major phospholipids, including major PC species, displayed comparable levels between genotypes in all time points analyzed (Figure 2C). In summary, we provide convincing evidence, obtained by 2 different mass spectrometry–based lipidomics approaches, implicating diabetes with a global reduction of myelin lipids in both the PNS and CNS that followed a typical “dying-back” neuropathy pattern, where longer and most distal peripheral nerve fibers are affected first and more dramatically.

### Myelin lipid losses precede changes in nerve structure/function.

**Figure 3:**
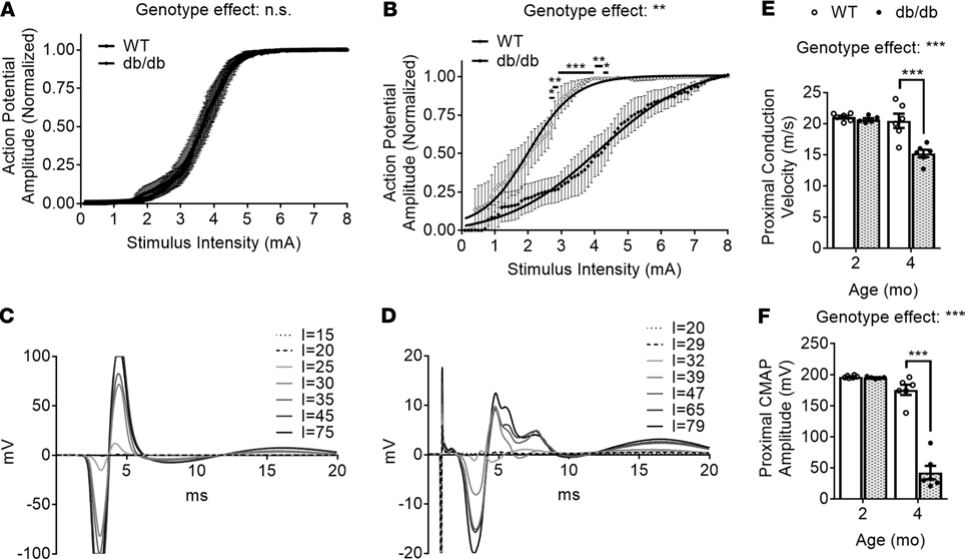
Impaired sciatic nerve function in obese diabetic mice. Relationship between the evoked action potential response and the stimulus intensity (input/output curves) for 2- and 4-month-old (**A** and **B**, respectively) WT (open circles/bars) and *db/db* (filled circles/bars) mice. Representative waveforms of sciatic nerve supramaximal responses in 4-month-old WT (**C**) and *db/db* (**D**) mice. Proximal conduction velocities (**E**) and compound motor action potential (CMAP) amplitudes (**F**) are displayed as dot blots with bars; each dot represents 1 animal; data are displayed as the mean ± SEM of *n* = 5–6 mice per genotype (left and/or right sciatic nerves were recorded 1–2 times for each mouse). Genotype effects for input/output curves were compared using 2-way ANOVA and Holm-Šidák multiple-comparisons tests on GraphPad Prism 7. *P < 0.05, **P < 0.01 ***P < 0.001.

Thanks to its high lipid-to-protein ratio, containing 70%–80% lipids versus 20%–30% proteins, myelin acts as an electrical insulator facilitating conduction in axons (32, 33). Thus, alterations in myelin lipid content are likely to result in myelin functional and/or structural changes. Following this rationale, we performed in vivo electrophysiological studies on obese diabetic mice. At early diabetic stages (2 months), action potentials and conduction velocities in *db/db* mice were indistinguishable from controls (Figure 3, A, E, and F). However, at more advanced diabetic stages (4 months), the nerves of *db/db* mice displayed a significant reduction of conduction velocities (Figure 3E), as well as a significant reduction in compound motor action potential (CMAP) amplitudes (Figure 3, B and F). WT sciatic nerves’ evoked responses showed clean biphasic waveforms with robust increases for each incremental stimulation before maximal responses were achieved (Figure 3C), while *db/db* sciatic nerves’ evoked responses were not biphasic but rather displayed multiple waveforms with weak increases for each incremental stimulation (Figure 3D). Taken together, our electrophysiology studies revealed a relatively late disruption of nerve function in *db/db* mice, both at the myelin (decreased conduction velocities) and axonal (decreased action potential amplitudes) levels, that occurred several months after myelin-enriched lipids were significantly decreased.

**Figure 4:**
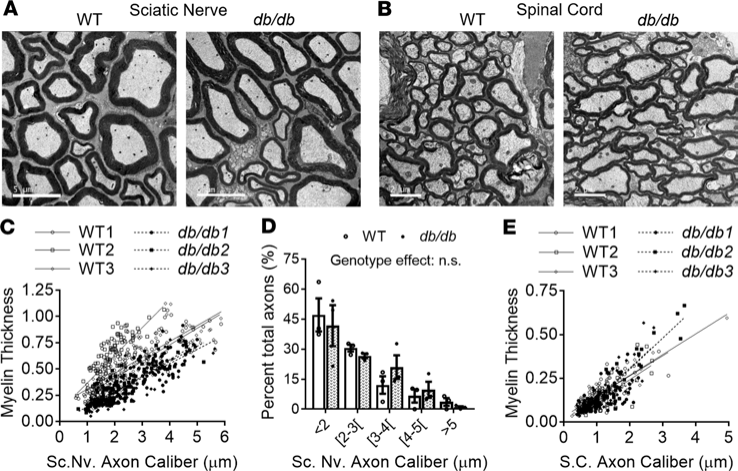
Disruption of myelin but not axonal PNS nerve ultrastructure in obese diabetic mice. Representative electron microscope (EM) cross-sectional images from the sciatic nerve (**A**) and spinal cord (**B**) (scale bars: 5 μm and 2 μm, respectively) of 4-month-old WT and *db/db* male mice. Scatter plots displaying myelin thickness of individual myelinated axons as a function of their respective average axon diameter (caliber) within the sciatic nerve (**C**) and spinal cord (**E**) in WT (open shapes and lines) and *db/db* (dotted shapes and broken lines) mice. Linear regression curves fitting the axonal data points (sciatic nerve *n* = ~100 axons/mice, spinal cord *n* = ~50 axons/mice) are displayed for each animal. (**D**) Sciatic nerve axons were categorized according to caliber; data are shown as the mean ± SEM of *n* = 3 male mice/genotype/tissue for all panels. Axon categories were compared between genotypes using 2-way ANOVA and Holm-Šidák multiple comparisons tests.

Finally, we assessed myelin ultrastructure via electron microscopy (EM) in the PNS and CNS of 4-month-old WT and *db/db* mice. Consistent with a reduction of myelin signature lipids, EM cross section studies revealed a significant decrease in average myelin thickness in the PNS (WT: 0.559 ± 0.017 μm, *db/db*: 0.357 ± 0.046 μm, *t* test *P* = 0.015) (Figure 4, A and C). On the other hand, average axon caliber was not significantly altered (WT: 2.32 ± 0.23 μm; *db/db*: 2.44 ± 0.29 μm, *t* test *P* = 0.77) between the 2 genotypes in the PNS (Figure 4, A and C). A significant genotype effect was observed for sciatic nerve g-ratio data (calculated as the diameter of the axon divided by the diameter of the entire myelinated fiber) (WT: 0.669 ± 0.028; *db/db*: 0.777 ± 0.004, *t* test *P* = 0.02). Further analyses, where PNS axons were categorized based on axon caliber, revealed that axonal size distributions were not significantly altered between both genotypes (Figure 4D). Although we did not find evidence of impaired ultrastructure at the axon diameter level (even at late diabetic stages), our electrophysiological results (reductions of action potential amplitudes) suggest that axon caliber may eventually be disrupted and/or that other aspects of axonal structure (at the paranodal and/or nodal level) may be altered in diabetic mice.

On the other hand, no significant differences were observed in the CNS (spinal cord) between the genotypes regarding myelin thickness or axon caliber (Figure 4, B and E), resulting in equivalent g-ratios (WT: 0.783 ± 0.015; *db/db*: 0.799 ± 0.006, *t* test *P* = 0.38). Again, these results are consistent with our lipidomics data as myelin lipid losses in the CNS were of later onset.

Taken together, our lipidomics, electrophysiological, and EM studies provide strong evidence that global losses of myelin lipids, which occur at early prediabetic stages in the PNS, precede any noticeable changes in nerve function/ultrastructure, which become evident at later stages of diabetes, exemplifying the power of lipidomics to detect very early disruptions of myelin homeostasis. Our results strongly suggest that at more advanced diabetic stages (uncontrolled diabetes), CNS nerve function/structure could also be eventually impaired.

### Early disruption of nerve mitochondrial lipids in db/db mice.

**Figure 5:**
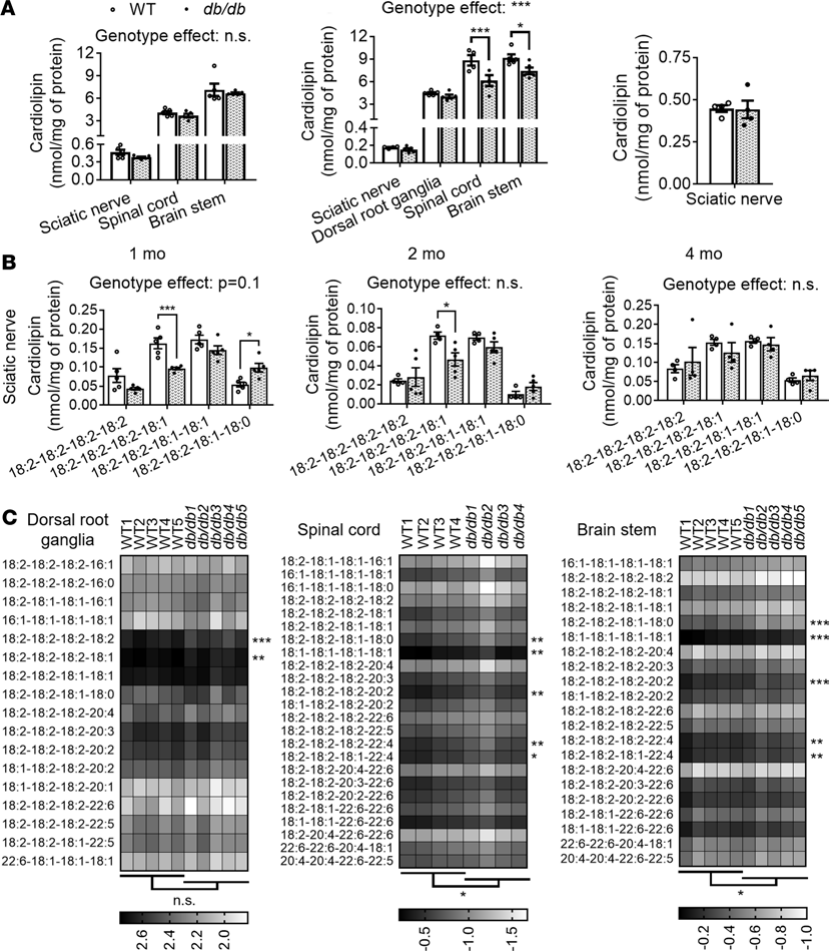
Impaired cardiolipin homeostasis in the PNS and CNS of obese diabetic mice. Sciatic nerve, DRG, spinal cord, and brainstem were dissected from 1-, 2-, and 4-month-old WT and *db/db* male mice, flash-frozen, homogenized, and lipid extracted. MDMS shotgun lipidomics was used to assess cardiolipin content (**A**) and composition (**B** and **C**) in different PNS and CNS regions. Graphs are presented as dot plots; data represent the mean ± SEM of *n* = 4–5 mice/genotype. WT (open circles/bars) and *db/db* (filled circles/bars). Cardiolipin molecular species for DRG, spinal cord, and brainstem of 2-month-old mice are presented as gray scale heatmaps (data were log transformed) (**C**). Low-abundance lipid species that made ≤1.5% of the total class content are not shown and were not included in statistical analyses. Although a specific mass peak may represent >1 cardiolipin molecular species, for simplicity heatmaps display only 1 lipid species (usually the most common) for each peak/row. Total cardiolipin levels were compared between genotypes for each region/time point using unpaired 2-tailed *t* tests. Molecular species within each lipid class were compared using 2-way ANOVA and Holm-Šidák multiple-comparisons test on GraphPad Prism 7. Statistics displayed below the heatmaps comparing genotypes and next to the molecular species are based on nontransformed data. *P < 0.05, **P < 0.01, ***P < 0.001.

Cardiolipin is almost exclusively found in the inner mitochondrial membrane, where it makes up about one-fifth of the total lipid content and plays essential structural and functional roles (34). This mitochondrial signature lipid interacts with a number of proteins and enzymes involved in fundamental mitochondrial bioenergetics, including respiratory chain complexes and mitochondrial substrate carriers, and is required for their optimal activity (35). Our lipidomics results revealed an early disruption of cardiolipin metabolism on nervous tissue of diabetic mice (Figure 5). Although no significant changes in the levels of total cardiolipin were observed between diabetic and control mice for any PNS or CNS regions at the earliest time point analyzed (1 month), a genotype effect trend was observed at the cardiolipin molecular species profile level in the sciatic nerve (Figure 5, A and B). In fact, 1 of the 2 major cardiolipin species (tri18:2-18:1), which makes up about one-third of total cardiolipin content under physiological conditions, was significantly reduced in diabetic nerves (–41%). It is important to note that healthy mitochondria are characterized by containing a high proportion of symmetrical (tetra18:2) or highly symmetrical (tri18:2) cardiolipin species (36, 37). In contrast, a less abundant cardiolipin species (di18:2-18:1-18:0), which under physiological conditions makes about 10% of total nerve cardiolipin content, was significantly increased in *db/db* nerves (+81%) compared with controls (Figure 5B). Asymmetrical cardiolipin species like this one have been linked with pathological states, mitochondrial dysfunction, and oxidative stress (38–40). It is important to mention that cardiolipin content in sciatic nerve is extremely low (1 order of magnitude lower than in CNS tissue).

At more advanced diabetic stages (2 months), a similar but milder cardiolipin remodeling was observed in the nerves of diabetic mice, including a significant decrease in cardiolipin tri18:2-18:1 (–35%) and an increasing trend in cardiolipin di18:2-18:1-18:0 (Figure 5B). Similarly, although total cardiolipin content in the DRG was also not significantly altered at this stage, 2 of the 3 most abundant cardiolipin species (tetra18:2 and tri18:2-18:1) were significantly reduced in the diabetic group (–15% and -23%, respectively) (Figure 5, A and C). Note that cardiolipin content in DRG is an order of magnitude higher than in nerves, consistent with higher mitochondrial densities in neuronal cell bodies versus axons. Finally, no significant genotype effects were observed in total sciatic nerve cardiolipin or in any of its molecular species at 4 months of age (Figure 5, A and B).

On the other hand, in CNS tissue no significant changes in total or specific cardiolipin species were observed between *db/db* and WT mice at 1 month (Figure 5A). However, spinal cord and brainstem tissue from 2-month-old *db/db* mice displayed a significant decrease of total cardiolipin content compared with controls (–31% and –19%, respectively) with several abundant cardiolipin species being significantly reduced (Figure 5, A and C). Taken together, our lipidomics data revealed that obese diabetic conditions influence cardiolipin metabolism in the PNS early on, affecting both nerve bundles and ganglia, and eventually affect the CNS as well. Unexpectedly, instead of becoming aggravated with disease progression, this initial disruption of nerve cardiolipin homeostasis seems to be eventually restored.

### Mitochondrial lipid losses precede changes in mitochondrial function.

**Figure 6:**
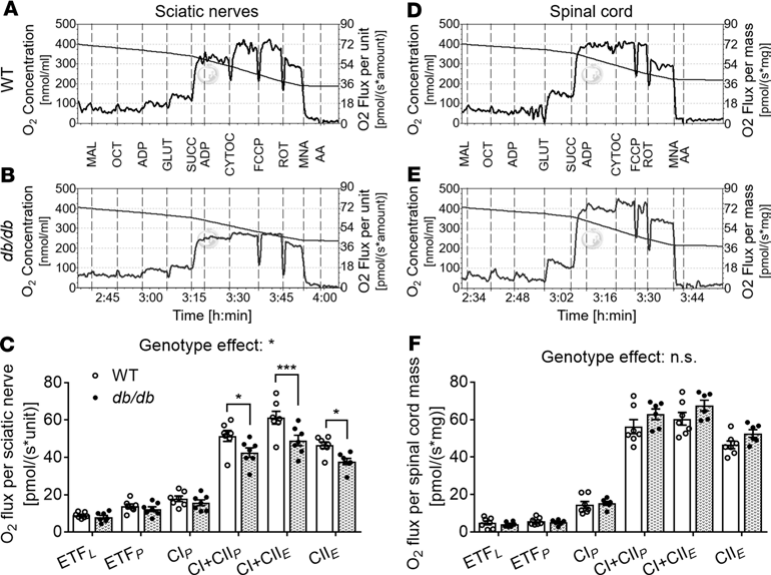
Reduced cellular respiration in sciatic nerve tissue of obese diabetic mice. Sciatic nerves (left and right) were permeabilized with collagenase, desheathed, and defluffed, while spinal cords were homogenized on a Kontes glass homogenizer in MiR06-Creatine respiration medium. High-resolution respirometry was conducted on Oxygraph-2K (Oroboros). Representative respirometric traces from sciatic nerve (**A** and **B**) and spinal cord (**D** and **E**) tissues using a substrate-uncoupler-inhibition titration (SUIT) protocol to measure oxygen consumption in WT (open circles/bars) and *db/db* (filled circles/bars) mice. Traces represent the oxygen concentration of the chamber (left *y* axis) and the oxygen flux per sciatic nerve unit or spinal cord mass (right *y* axis). Respiration analyses for sciatic nerve (**C**) and spinal cord tissue (**F**) are graphically represented as dot plots with bars; each dot represents 1 animal; data are displayed as the mean ± SEM of *n* = 6–7 male mice/genotype/tissue. Respiration parameters were compared between genotypes using 2-way ANOVA and Holm-Šidák multiple-comparisons tests on GraphPad Prism 7. *P < 0.05, ***P < 0.001. “Genotype effect” describes the global 2-way ANOVA results (considering all the measurements). The *P* value markers shown above brackets are from the multiple comparisons done between genotypes for each individual measure. ETF*_L_* (fatty acid oxidation in the absence of ADP [state 2]); ETF*_P_* (fatty acid oxidation coupled to ATP production); CI*_P_* (complex I-linked respiration coupled [state 3]); CI + CII*_P_* (complex I and complex II-linked respiration coupled [state 3]); CI + CII*_E_* (complex I and complex II-linked respiration uncoupled [maximum respiration]); and CII*_E_* (complex II activity uncoupled). Experimental mice were 2–2.5 months old. MAL, malate; OCT, octanoylcarnitine; GLUT, glutamate; SUCC, succinate; CYTOC, cytochrome c; FCCP, carbonylcyanide *p*-trifluoromethoxyphenylhydrazone; ROT, rotenone; MNA, malonic acid; AA, antimycin A.

Because altered cardiolipin homeostasis is strongly indicative of impaired mitochondrial function (38), we proceeded to examine mitochondrial bioenergetics via high-resolution respirometry (HRR) in sciatic nerve and spinal cord tissue of WT and *db/db* mice. To this end, we quantified oxidative phosphorylation in response to specific substrates for complex I (CI), complex II (CII), fatty acid oxidation (FAO), and electron transfer system capacity following a substrate-uncoupler-inhibition titration (SUIT) protocol (described in the Methods section). We found a significant genotype effect for oxygen flux in sciatic nerve tissue at 2 months of age, indicating that global respiratory capacity was significantly impaired in *db/db* mice compared with WT controls (Figure 6). Specifically, we observed that whole *db/db* sciatic nerves displayed a significant decrease in maximal uncoupled respiration (–23%), defined as the electron transfer system (ETS) capacity reached in the presence of the uncoupler FCCP (CI + CII*_E_*, where E denotes ETS); a significant decrease in CII uncoupled respiration (–25%, *q* = 0.046), defined as succinate-supported ETS capacity (CII*_E_*) in the presence of rotenone, a CI inhibitor; and a significant decrease in maximal ADP-saturated coupled respiration (–21%), defined as CI+CII-linked oxidative phosphorylation (OXPHOS) capacity (CI + CII*_P_*) (Figure 6, A–C).

On the other hand, fatty acid oxidation (FAO) through the electron transferring flavoprotein (ETF) complex in the presence (coupled, ETF*_P_*) or absence of ADP (uncoupled, ETF*_L_*, where *L* denotes leak respiration) was not significantly altered between genotypes. Likewise, no genotype effect was observed for coupled CI respiration (CI-linked OXPHOS capacity, or CI*_P_*) (Figure 6, A–C); thus, the described defects seem to be primarily driven by impaired CII respiration. It is important to note that the tissue weights of equally dissected sciatic nerves (i.e., equal lengths) tended to be reduced in diabetic mice compared with controls (–10%). This decrease in tissue weight is likely due (at least partially) to reduced myelin content. After normalizing oxygen flux measures to sciatic nerve weight, none of them were significantly affected by genotype (data not shown).

No significant genotype effect on global respiratory capacity was observed on spinal cord tissue in any of the respiration states analyzed at 2 months of age (Figure 6, D–F). It is important to note that cytochrome c was added to test the integrity of the outer mitochondrial membrane (41). The increase of oxygen flux with cytochrome c was on average (including both genotypes as there was no genotype effect) 7.7% ± 0.49% and 7.1% ± 0.81% of maximum respiration in sciatic nerve and spinal cord tissue, respectively, demonstrating that mitochondrial integrity was well preserved during our experimental procedure (41). Taken together, our mitochondrial respiration and lipidomics data demonstrate that cardiolipin remodeling in the nerves of obese diabetic mice precedes and is associated with a mild impairment in mitochondrial function.

### Mitochondrial/myelin lipid losses associate with abnormal mitochondrial/myelin protein content.

**Figure 7:**
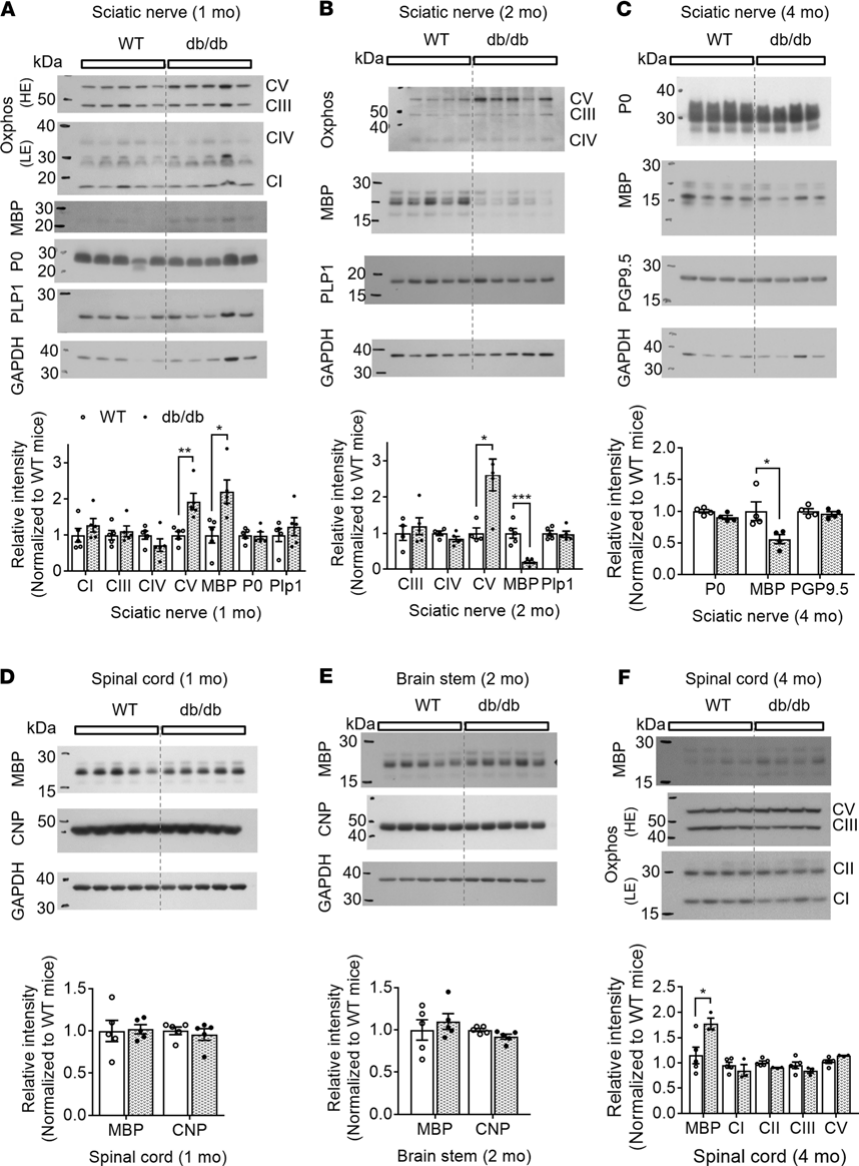
Alterations in specific mitochondrial and myelin-specific proteins in obese diabetic mice. Sciatic nerve, spinal cord, and/or brainstem NP-40 homogenates (supernatants) from 1-month-old (**A** and **D**), 2-month-old (**B** and **E**), and 4-month-old (**C** and **F**) WT and *db/db* mice. Representative Western blots using oxidative phosphorylation (OXPHOS) rodent antibody cocktail, as well as antibodies against proteolipid protein 1 (PLP1), myelin basic protein (MBP), P0 (myelin protein zero, MPZ), and cyclic-nucleotide phosphodiesterase (CNP). Relative intensities (normalized to WT mice) were quantified using ImageJ software. GAPDH is shown as an example of a loading control. Relative intensities for each protein were compared between genotypes using unpaired 2-tailed Student’s *t* tests. Graphs are presented as dot plots with bars; each dot represents a different animal; data represent the mean ± SEM of *n* = 4–5 mice/genotype for all panels. *P < 0.05, **P < 0.01. Full, unedited versions of the gels are provided in the supplemental material.

Finally, seeking to gain further mechanistic insights regarding diabetes-related myelin and mitochondrial dysfunction, we analyzed the levels of major mitochondrial OXPHOS and myelin proteins in PNS/CNS tissue from diabetic mice. Western blot analysis using OXPHOS antibody cocktail revealed an early and specific genotype effect in sciatic nerve complex V (CV, ATP synthase) content. Surprisingly, CV was significantly increased (+93%) in *db/db* nerves at the earliest time point analyzed (1 month) (Figure 7A). This increase in CV became more dramatic with age (+160% in 2-month-old *db/db* sciatic nerve tissue). On the other hand, this increase in CV was not evident in the CNS (Figure 7B).

Conversely, none of the major myelin proteins were significantly decreased at the earliest time point analyzed (1 month) for *db/db* mice. Paradoxically, the abundance of total myelin basic protein (MBP), a major myelin protein, was significantly increased (+120%) (Figure 7A). However, at 2 months of age, we observed a dramatic reduction (–79%) of MBP in *db/db* sciatic nerves compared with controls (Figure 7B). Significant decreases in MBP levels were also observed in sciatic nerves of 4-month-old *db/db* mice (–44%) (Figure 7C). Similarly, we observed a significant reduction of MBP levels in the nerves of HFD-fed mice compared with their respective controls (–34%) (Supplemental Figure 6). The levels of myelin protein zero (MPZ), the major PNS myelin protein, were not altered in any of the obese (pre)diabetic models/time points/tissues analyzed (Figure 7 and Supplemental Figure 6). Furthermore, in the CNS, we did not observe reductions in the levels of any of the major myelin proteins analyzed, including MBP and cyclic-nucleotide phosphodiesterase (CNP), in any of the models/time points/tissues analyzed (Figure 7, D–F). MBP was actually significantly increased in the spinal cord of 4-month-old *db/db* mice (Figure 7F), reminiscent of what was observed in the PNS nerves at very early stages. Based on these results, it seems reasonable to predict that at more advanced diabetic stages, MBP may also be eventually decreased in the CNS of *db/db* mice. We also assessed a pan-neuronal marker in the PNS; however, no significant differences were observed in the levels of PGP9.5 in sciatic nerve tissue of diabetic mice at the latest time point examined (Figure 7C).

### Myelin losses in diet-induced prediabetic obese mice.

Next, we were interested in studying if the losses of mitochondrial and myelin lipids observed in *db/db* mice, where leptin receptor deficiency induces diabetes, also occurred in diet-induced obese mice. We used a long-term (9 months) high-fat diet (HFD) regimen (60% kcal fat) on C57BL/6J mice as a model of prediabetes obesity. HFD-fed mice developed severe obesity (+71% in body weight), hyperinsulinemia (+2.9-fold in nonfasted serum insulin), and hypertriglyceridemia (+53% in nonfasted serum triacylglyceride, TAG) but did not display basal hyperglycemia (no significant changes in nonfasted blood glucose) (Supplemental Figure 7).

**Figure 8:**
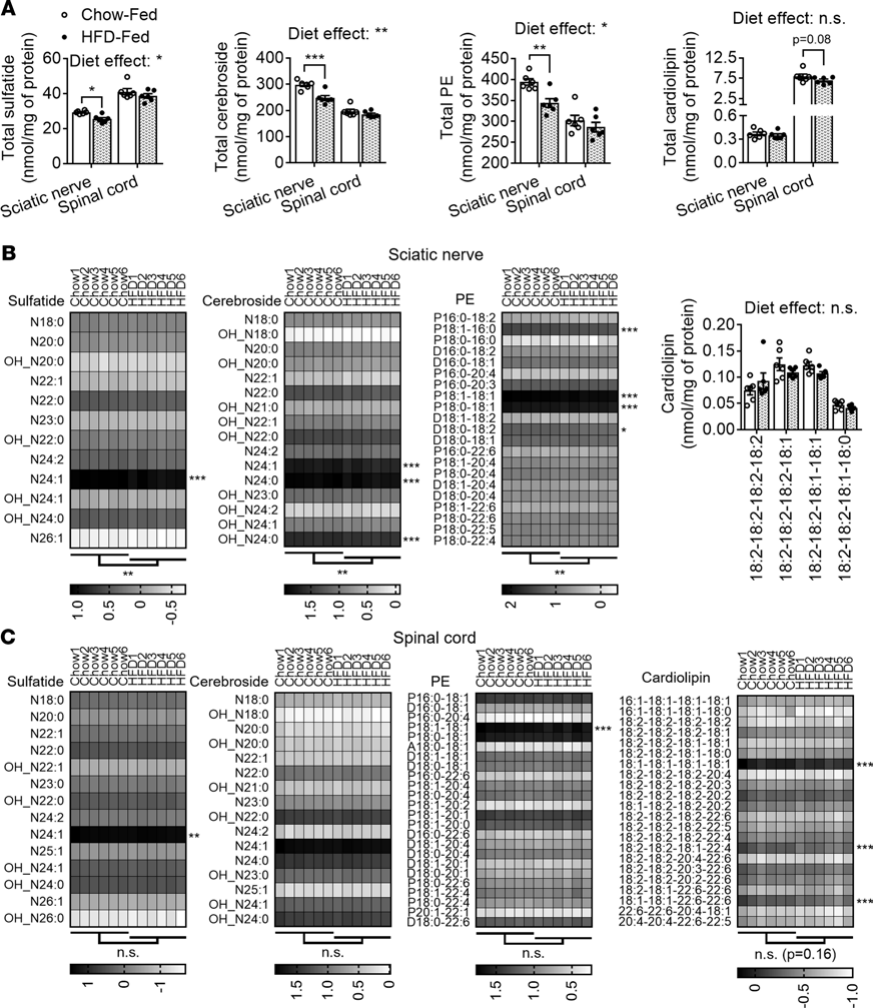
Loss of myelin lipids in diet-induced obese prediabetic mice. Sciatic nerve and spinal cord tissue from long-term HFD-fed and chow-fed controls were dissected, flash-frozen, homogenized, lipid extracted, and assessed by MDMS shotgun lipidomics. (**A**) Total sulfatide, cerebroside, and PE, and cardiolipin content for each dietary regimen/tissue is displayed as dot plots (chow shown with open circles/bars, HFD shown with filled circles/bars); data represent the mean ± SEM of *n* = 6 mice/dietary regimen for all panels. Total levels of each lipid class were compared between dietary regimens and tissues using 2-way ANOVA (genotype effect *P* values specified on top of each graph) and Holm-Šidák multiple-comparisons tests (adjusted *P* values specified on top of brackets). Sciatic nerve (**B**) and spinal cord (**C**) molecular species of each lipid class mentioned above are displayed as either dot blots with bars (sciatic nerve cardiolipin) or log-transformed gray scale heatmaps (all others). Low-abundance lipid species that made ≤1% of the total class content are not shown and were not included in statistical analyses. Although a specific mass peak may represent more than one molecular species, for simplicity heatmaps display only 1 (most common) lipid species for each mass (row). Molecular species within each lipid class were compared using 2-way ANOVA and Holm-Šidák multiple-comparisons tests on GraphPad Prism 7. Statistics displayed below the heatmaps represent global comparisons between dietary regimens, while those next to specific rows represent specific molecular species comparisons. *P < 0.05, **P < 0.01, ***P < 0.001.

Lipidomics analyses revealed a significant reduction of total sulfatide (–13%), cerebroside (–17%), and PE content (–13%) in sciatic nerve tissue of HFD-fed mice compared with chow-fed controls (Figure 8A). Again, the most abundant species of these myelin-enriched classes were significantly reduced (Figure 8B). On the other hand, spinal cords from HFD-fed mice did not display any significant alterations in total sulfatide, cerebroside, or PE content (Figure 8A). Although detailed analyses of specific molecular species did not reveal a significant group effect (between dietary regimens) at the CNS level, some major species of each of these myelin-enriched lipid classes were significantly decreased in obese prediabetic mice (Figure 8C). The fact that global myelin lipid losses in the nervous system of HFD-fed mice were lower in magnitude compared with *db/db* mice is consistent with their overall milder (pre)diabetic pathology.

Finally, no significant alterations were observed in total cardiolipin content between the 2 feeding regimens in the PNS or CNS (Figure 8A). Detailed analysis of cardiolipin molecular species did not reveal significant group effects either (Figure 8, B and C). However, several cardiolipin species (including the most abundant one) were significantly decreased in the CNS of HFD-fed mice (Figure 8C), reminiscent of the significant disruption of cardiolipin homeostasis observed early on in *db/db* mice. Perhaps we did not observe cardiolipin remodeling in the PNS because these mice had been on HFD for a long period, as this early phenotype was eventually restored in *db/db* mice.

### Hyperglycemia does not drive early nerve mitochondrial/myelin lipid losses.

Next, we assessed streptozotocin-induced (STZ-induced) diabetes on C57BL/6J mice using 2 different protocols where animals were subjected to insulin-deficient hyperglycemic conditions for short-term (10 days) and long-term (2.5 months) periods. The short-term group received a single high dose of STZ (shown to exert direct toxicity on insulin-producing cells, leading to necrosis within 2–3 days, ref. 42) that led to hypoinsulinemia (–58%), overt basal (nonfasted) hyperglycemia (+4-fold), and a mild loss of body weight (–18%) (Supplemental Figure 8A). The long-term group received multiple low doses of STZ administered for 5 consecutive days (shown to induce both β-cytotoxic effects and STZ-specific, T cell–dependent immune reactions) that led to overt hypoinsulinemia, gradual basal hyperglycemia, and significant loss of body weight (Supplemental Figure 7B). On the other hand, neither “high-dose short-term” nor “low-dose long-term” STZ treatment led to significant alterations of total serum free fatty acid (FFA) or TAG under basal (nonfasted) conditions (Supplemental Figure 8, A and B).

**Figure 9:**
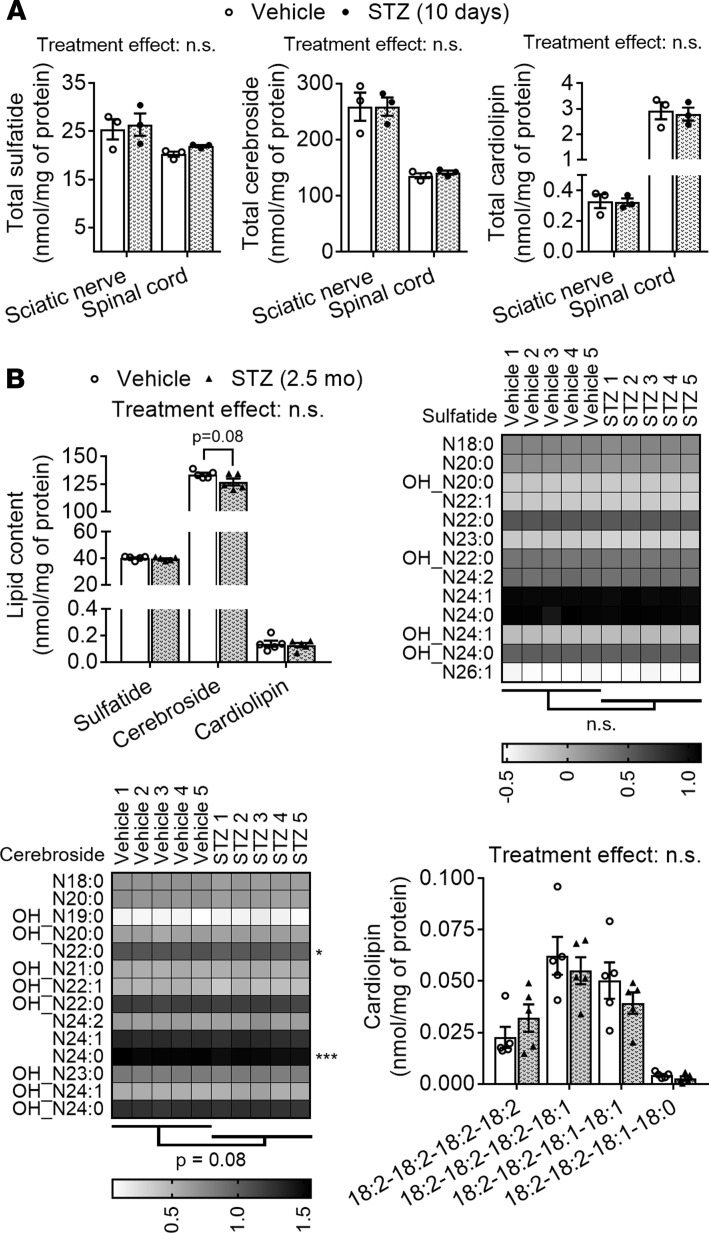
Insulin deficiency–induced hyperglycemia does not alter nerve mitochondrial/myelin lipid homeostasis. Sciatic nerve and spinal cord tissue from vehicle and insulin-deficient STZ-treated mice were lipid extracted and assessed by shotgun lipidomics. Tissue was harvested 10 days after a single high dose of STZ (filled circles/bars) or vehicle (open circles/bars (**A**) or 2.5 months after multiple low doses of STZ or vehicle (**B**). Total sulfatide, cerebroside, and cardiolipin content in sciatic nerve and spinal cord tissue from high-dose short-term STZ-treated mice. Total sulfatide, cerebroside, and cardiolipin content in sciatic nerves from low-dose long-term STZ-treated mice. Detailed analyses of sulfatide, cerebroside (CBS) and cardiolipin molecular species. Low-abundance lipid species that made ≤1% of the total class content are not shown and were not included in statistical analyses. In addition, very low-abundance sciatic nerve cardiolipin molecular species with mass levels ≤ 0.01 nmol/mg of total protein content were excluded. Although a specific mass peak may represent >1 molecular species, for simplicity heatmaps display only the most common lipid species for each peak (row). Graphs are presented as dot plots with bars; each dot represents 1 animal; data represent the mean ± SEM of *n* = 3 and 5 mice/group. Molecular species are presented as log-transformed gray scale heatmaps. Molecular species within each lipid class were compared using 2-way ANOVA and Holm-Šidák multiple-comparisons tests on GraphPad Prism 7. Statistics displayed below the heatmaps compare treatments and next to the molecular species are based on nontransformed data. *P < 0.05.

Surprisingly, none of the STZ treatments led to significant alterations in total content of myelin or mitochondrial signature lipids in PNS or CNS tissue (Figure 9). Specifically, total levels of sulfatide and cerebroside were not significantly altered at either dose in sciatic nerve tissue or in spinal cord (Figure 9). Detailed analyses of specific sulfatide and cerebroside species revealed that most of them were not significantly altered, although a couple of cerebroside species were mildly decreased (–11% to –13%) in the nerves of low-dose long-term STZ-treated nerves (Figure 9B). Moreover, total cardiolipin content was also unaltered in the PNS or CNS of STZ-treated mice (Figure 9A). Detailed analysis of cardiolipin species revealed that none of them were significantly altered in STZ-treated mice (Figure 9B).

### Hyperinsulinemia or systemic insulin resistance does not drive early nerve mitochondrial/myelin lipid losses.

Finally, we assessed a model of nonobese diabetes, i.e., transgenic mice expressing a mutant dominant-negative insulin-like growth factor-1 receptor (KR-IGF-1R) specifically targeted to skeletal muscle (MKR mice) (43). MKR mice displayed early and progressive basal hyperglycemia compared with WT (FVB/N) controls (+44% and +76% at 1 and 2 months, respectively) that eventually diminished at advanced stages (+26% at 4 months) (Supplemental Figure 9B). In addition, MKR mice displayed early, severe, and progressive hyperinsulinemia that progressed with age and a significant and progressive loss of body weight (–10 to –11% at 1–2 months; –18% at 4 months) (Supplemental Figure 9A). All of the abovementioned results are consistent with those reported in the literature for this mouse line (43, 44). On the other hand, MKR mice did not display significant alterations of total serum FFA or TAG under basal conditions at any of the time points analyzed (Supplemental Figure 9, C and D).

**Figure 10:**
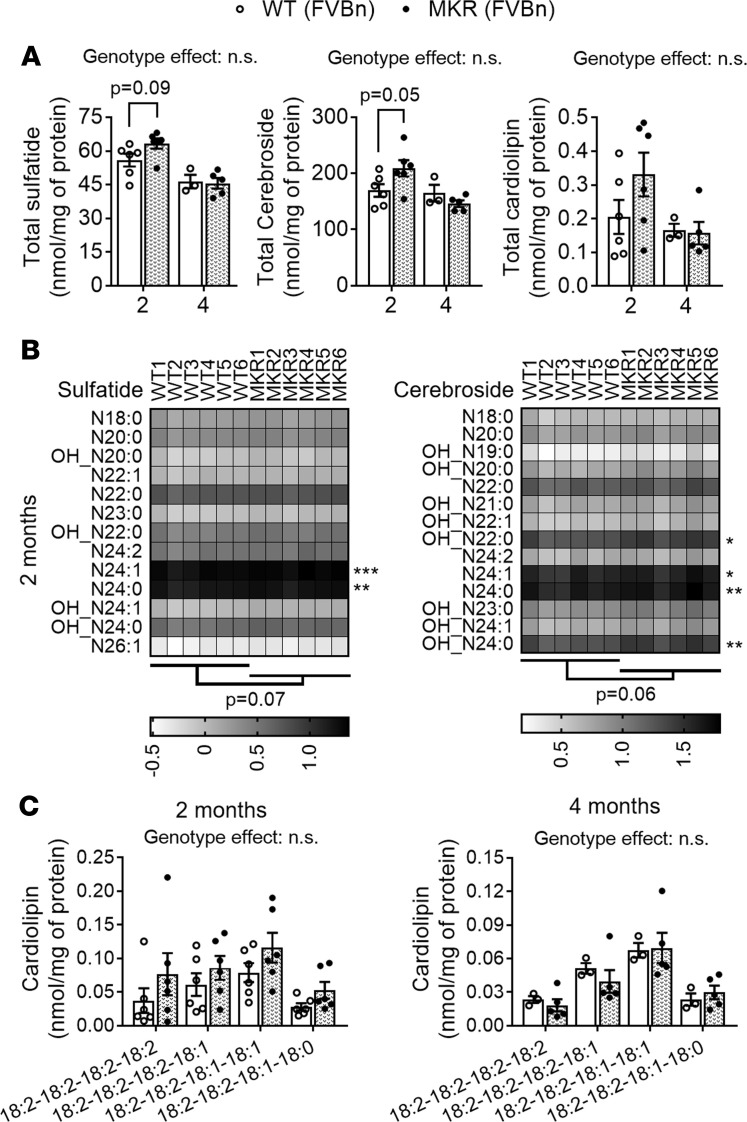
Hyperinsulinemia and insulin resistance do not alter nerve mitochondrial/myelin lipid homeostasis. Sciatic nerve tissue from WT (open circles/bars) and MKR (filled circles/bars) mice (both on an FVB/n genetic background) was lipid extracted and assessed by shotgun lipidomics. (**A**) Total sulfatide, cerebroside, and cardiolipin content from 2- and 4-month-old mice. (**B**) Sulfatide and cerebroside (CBS) molecular species from sciatic nerves of 2-month-old mice. (**C**) Cardiolipin molecular species from sciatic nerves of 2- and 4-month-old mice. Total levels of each lipid class are displayed as dot plots (WT: open circles/bars; MKR: filled circles/bars); data represent the mean ± SEM of *n* = 6 mice/genotype. Total levels of each lipid class were compared between genotypes for every tissue examined at each time point using 2-way ANOVA (genotype effect *P* values specified on top of each graph) and Holm-Šidák multiple-comparisons tests (adjusted *P* values specified on top of brackets). Molecular species masses expressed as nmol/mg of protein were log transformed and displayed as gray scale heatmaps. Low-abundance lipid species that made ≤1% of the total class content are not shown and were not included in statistical analyses. Although a specific mass peak may represent >1 cardiolipin molecular species, for simplicity heatmaps display only 1 (most common) lipid species for each mass (row). Molecular species within each lipid class were compared using 2-way ANOVA and Holm-Šidák multiple-comparisons tests on GraphPad Prism 7. Statistics displayed below the heatmaps comparing genotypes and next to the molecular species are based on nontransformed data.

Intriguingly, MKR mice did not display significant losses of myelin or mitochondrial signature lipids in the PNS (Figure 10). Total sciatic nerve sulfatide and cerebroside levels were not significantly altered even at advanced nonobese diabetic stages (4 months) and if anything tended to increase at midstages (2 months) (Figure 10A). Detailed lipidomic analyses revealed that all sulfatide and cerebroside species were unaltered at 4 months of age (data not shown), while at 2 months of age a few species were actually significantly increased in sciatic nerve tissue of MKR relative to WT controls (Figure 10B). Moreover, total sciatic nerve cardiolipin levels were also unaltered (Figure 10A), with none of the specific cardiolipin species being significantly altered either (Figure 10C). Although we cannot exclude the possibility that even longer periods of hyperinsulinemia and/or insulin resistance could eventually lead to global losses of nerve mitochondrial/myelin lipids, it seems clear that hyperglycemia, hyperinsulinemia, and insulin are not the main drivers of the early disruption of nerve lipid homeostasis observed in obese (pre)diabetic animals.

## Discussion

### Obesity-induced prediabetes is associated with global losses of myelin lipids that are not driven by hyperglycemia, dysinsulinemia, or insulin resistance.

To the best of our knowledge, this study is the first to demonstrate a global loss of myelin lipids during early stages of obesity-associated diabetes at a preclinical level. Importantly, we provide robust evidence supporting this statement, including data from multiple PNS and CNS regions from 2 preclinical models of obesity-associated (pre)diabetes (*db/db* and HFD-fed mice), as well as 2 models of nonobese diabetes (STZ-treated and MKR mice), using 2 mass spectrometry–based methodologies (MDMS shotgun lipidomics and MALDI imaging). Spatiotemporal analyses revealed that disruption of myelin lipid maintenance in obese (pre)diabetic mice preceded pathogenic myelin changes at the protein, ultrastructure, and functional levels. The progression of this global loss of myelin lipids resembled the typical pattern of dying-back axonal degeneration or distal axonopathy characterized by progressive peripheral brain nerve damage, where long peripheral sensorimotor distal nerve terminals are affected first while proximal neuronal somas are affected last (45, 46). Following this pattern, lipid homeostasis in diabetic sciatic nerves was severely affected early on, while DRG were mildly affected later on. Similarly, abnormal lipid profiles occurred earlier and more dramatically in the PNS compared with the CNS.

The early onset of this disruption of myelin lipid homeostasis in *db/db* mice was remarkable and consistent with the premature/aggressive nature of this genetic model. The described nerve lipid abnormalities also occurred in diet-induced obese mice, where HFD feeding did not start until 3 months of age, when mice have reached mature adulthood, and their PNS/CNS has been fully developed, ruling out the possibility that the observed myelin/mitochondrial phenotypes occur as a developmental adaptation to pathological conditions.

On the other hand, nonobese diabetic mouse models displayed normal myelin lipid homeostasis. Surprisingly, overt hyperglycemia induced by STZ treatment had virtually no effect on nerve mitochondrial or myelin lipid homeostasis, even after prolonged periods of time. Similarly, nonobese diabetic MKR transgenic mice displayed physiological nerve mitochondrial/myelin lipid levels even at advanced diabetic stages, despite the fact that they also displayed early basal hyperglycemia. Similarly, neither overt hypoinsulinemia in STZ mice nor early/severe hyperinsulinemia in MKR mice disrupted nerve mitochondrial/myelin lipid homeostasis. Considering that MKR mice develop early and severe whole-body insulin resistance (43, 44) and that STZ treatment has been shown to induce progressive insulin resistance in peripheral tissues (47, 48), insulin resistance does not seem to be sufficient to drive the observed losses of nerve mitochondrial/myelin lipids in obese (pre)diabetic mice.

Taken together, our results provide strong evidence that the disruption of nerve mitochondrial/myelin lipid homeostasis observed in obese (pre)diabetic mice is not causally linked to hyperglycemia, dysinsulinemia, or insulin resistance but rather seems to be primarily driven by other components of the metabolic syndrome, namely obesity and/or hyperlipidemia. Consistent with this notion, a high prevalence of peripheral neuropathy has been reported in severely obese nondiabetic patients (49–52). Moreover, an excess of dietary saturated fatty acids has been shown to be sufficient to drive peripheral nerve damage by impairing mitochondrial function and motility in dorsal root ganglion neurons (53, 54). Further supporting this notion, high saturated fatty acid content has been associated with decreased cardiolipin synthesis in other organs (55). Similarly, fatty acid transport has been proposed to regulate cardiolipin biosynthesis (56). Finally, clinical studies have concluded that obesity and hyperlipidemia are strongly associated with diabetic neuropathy (50, 57, 58), even under nondiabetic conditions (49, 59), while epidemiological and preclinical studies on the etiology of peripheral neuropathies have proposed hyperlipidemia as a novel risk/causal factor (12–16, 60–62).

The novelty of this study relies on (a) our focus on peripheral nerves and myelin in addition to assessing the neuronal cell body–rich DRG, (b) our lipidomics approach that led to the discovery of potentially novel lipid biomarkers/therapeutic targets to diagnose/treat a new subtype of peripheral neuropathy associated with obesity, and (c) our multimodel approach to compare the effects of different components of the metabolic syndrome on the nerve lipidome. Notably, this study exemplifies the high sensitivity of lipid metabolism to pathological conditions and demonstrates the power of functional lipidomics to better understand disease etiology and/or to discover novel disease biomarkers.

### Molecular mechanisms leading to depletion of myelin lipids.

The described disruption of mitochondrial and myelin lipid homeostasis characteristic of obesity-induced diabetes does not seem to occur as a consequence of a global loss of function of lipid biosynthesis machinery because the expression of fatty acid synthesis–related genes was not decreased. Furthermore, the fact that major cellular phospholipid classes (e.g., PC) were unaltered points to a specific loss of mitochondrial/myelin signature lipids.

Given that mitochondrial abnormalities co-occurred with myelin damage, it is tempting to speculate that energy substrate shortage and/or redistribution within myelinating cells leads to impaired myelin maintenance due to a shortage of ATP and/or energy metabolites, some of which are essential for lipid synthesis (e.g., citrate, acetate). This idea is strongly supported by the early increases in OXPHOS CV (ATPase) and initial cardiolipin deficiency in obese diabetic nerves indicative of altered bioenergetics. It is important to note that these mitochondrial abnormalities, besides occurring within nerve bundles/myelinating cells, also affect neuronal cell bodies in the DRG, as supported by our data and previous reports (53, 54, 63). Moreover, hyperlipidemia is almost certainly associated with impaired FAO/abnormal energy substrate usage. The association of hyperlipidemia with nerve myelin damage also opens the possibility that increased FFAs result in lipotoxicity-induced mitochondrial/neuronal/myelin damage.

The “dying-back” phenomenon resemblance strongly suggests that distal myelinating Schwann cells are particularly vulnerable under obese (pre)diabetic conditions. As Schwann cells are intimately associated with the axon and respond dynamically to axon injury (64, 65), the observed loss of myelin lipids could be explained by atrophy of Schwann cells myelinating distal damaged axons. This possibility is supported by multiple reports describing early losses of intraepidermal nerve fiber (IENF) density in *db/db* mice (66–68) and by the fact that IENF axons are primarily unmyelinated. However, the converse mechanism where distal Schwann cell atrophy drives terminal axonal degeneration is also plausible. In fact, terminal nonmyelinating Schwann cells help maintain the physical integrity of the synaptic junction. Our EM studies on obese diabetic mice support the possibility that disruption of Schwann cell homeostasis precedes axonal damage, as ultrastructural myelin abnormalities were evident before any noticeable disruption of axonal ultrastructure.

Taken together our results add to the controversy as to whether Schwann cell atrophy and/or neuronal/axonal damage are the primary lesions driving diabetic neuropathy. Our data prompted us to propose a model where obesity/hyperlipidemia induces nerve damage preferentially by affecting glial cells, while neurons (terminal axons) are likely more prone to hyperglycemia-induced nerve damage. Although distinguishing between these possibilities falls outside of the scope of this report, future elaborate mechanistic studies are needed to better understand the underlying molecular mechanisms causally linking hyperglycemia, obesity, hyperlipidemia, Schwann cell/neuronal homeostasis, and nerve function.

### Sulfatide deficiency is a potentially novel molecular mechanism linking diabetes and Alzheimer’s disease.

Although obese diabetic mice displayed a global loss of myelin lipids, it is important to note that sulfatide was typically the first myelin-enriched lipid class to be significantly impaired throughout our spatiotemporal studies on diabetic mice. Interestingly, we have also shown that sulfatide content is dramatically reduced in the brain in Alzheimer’s disease (AD) (69–71). Considering that diabetes has been established as a risk factor for AD (7–11), we propose brain sulfatide deficiency as a novel molecular mechanism that could potentially link advanced diabetes with AD. This model is supported by the results presented here demonstrating that although diabetes-associated sulfatide deficiency initiates in the periphery, it can eventually progress to the brain at advanced disease stages, at which point it could contribute to AD pathogenesis by accelerating brain sulfatide deficiency, a process that in the context of AD is mediated by apolipoprotein E and accelerated by amyloid-β (72).

## Methods

### Mouse models.

Mice homozygous for the diabetes spontaneous mutation *Lepr^db^* (*db/db*) and WT mice in a C57BL/6J background were purchased from The Jackson Laboratory (JAX), 000697 and 000664, respectively. Mice were provided with food and water ad libitum and were euthanized at 1, 2, and 4 months (*n* = 10–16 male mice per genotype per time point). An additional cohort of JAX C57BL/6J WT male mice were fed with chow or HFD (60% kcal fat, Research Diets Inc., catalog D12492), starting at 3 months of age, and were euthanized at 12 months (*n* = 10–13 male mice per feeding regimen).

Additional cohorts of JAX C57BL/6J WT mice were injected with STZ or with control saline buffer following 2 different protocols involving different STZ doses (high and low) and postinjection periods (short and long term). The high-dose short-term protocol consisted of a single IP injection of 125 mg STZ/kg body weight or saline and a 10-day postinjection period. The low-dose long-term protocol consisted of daily IP injections of 50 mg STZ/kg body weight or buffer only for 5 consecutive days and a 2.5-month postinjection period. This last protocol was performed by The Jackson Laboratory services following their validated protocol that involves treating 2-month-old mice and shipping them 18 days postinjection (*n* = 5 male mice per treatment).

MKR (in an FVB/n genetic background) and FVB/n WT control founder mice were provided by Derek LeRoith’s laboratory (Icahn School of Medicine at Mount Sinai, New York, New York, USA) (43). MKR mice were bred in University of Texas Health San Antonio (UT Health SA) animal facility, provided with food and water ad libitum, and euthanized at 2 and 4 months of age (*n* = 8–15/genotype/time point). Body weights and blood glucose levels were measured at 1, 2, and 4 months.

### Tissue harvest/preparation.

Blood and nervous tissues (cerebrum, brainstem, spinal cord, and sciatic nerve) were harvested for all animal cohorts described above. Fluid/tissues were either flash-frozen in liquid nitrogen for biochemical studies or processed immediately for physiological studies. Frozen tissues were weighted, lyophilized for 24 hours, pulverized, and homogenized on a cooling bead beater (Cryolys Precellys Evolution Homogenizer) for the biochemical studies described below.

### Blood glucose and insulin measurements.

Right before euthanasia, mouse tails were clipped and blood glucose was measured using a glucose meter (Contour, Bayer). Serum insulin was assessed from blood plasma using Mercodia mouse insulin ELISA following the manufacturer’s instructions. Maximum levels of detection were used for samples that fell above the glucose meter range or insulin calibration curve.

### MDMS shotgun lipidomics.

Cerebrum, brainstem, spinal cord, and sciatic nerve tissues were homogenized in ice-cold diluted phosphate-buffered saline (0.1× PBS) as described previously (73). Lipids were extracted by a modified procedure of Bligh and Dyer extraction in the presence of internal standards, which were added based on the total protein content of individual samples, as described previously (74–76).

A triple-quadrupole mass spectrometer (Thermo Fisher Scientific TSQ Altis) and a Quadrupole-Orbitrap mass spectrometer (Thermo Q Exactive) equipped with a Nanomate device (Advion Bioscience Ltd.) and Xcalibur system software was used as previously described (77–79). Briefly, diluted lipid extracts were directly infused into the ESI source through a Nanomate device. Typically, signals were averaged over a 1-minute period in the profile mode for each full-scan MS spectrum. For tandem mass spectrometry (MS), a collision gas pressure was set at 1.0 mTorr, but the collision energy varied with the classes of lipids. Similarly, a 2- to 5-minute period of signal averaging in the profile mode was employed for each tandem MS mass spectrum. All full and tandem MS mass spectra were automatically acquired using a customized sequence subroutine operated under Xcalibur software. Data processing, including ion peak selection, baseline correction, data transfer, peak intensity comparison, ^13^C deisotoping, and quantitation, were conducted using a custom-programmed Microsoft Excel macro as previously described after considering the principles of lipidomics (78, 79).

### Gene expression analyses.

Total RNA was isolated after homogenizing sciatic nerves in Invitrogen TRIzol reagent (Thermo Fisher Scientific) using Direct-zol RNA MiniPrep kit for RNA isolation (Zymo Research, catalog R2050). RNA concentration was quantified using a NanoDrop 8000 spectrophotometer (Thermo Fisher Scientific). mRNA was reverse-transcribed from 800 ng of total RNA using Applied Biosystems High-Capacity cDNA Reverse Transcription Kit (Thermo Fisher Scientific, catalog 4368814). Quantitative reverse transcription PCR was performed using SsoFast EvaGreen Supermix (Bio-Rad) and a 2-step program (60°C annealing temperature) on a LightCycler 480 Instrument II (Roche). L32 expression was used to normalize samples and obtain relative expression. Primer sequences used are provided in Supplemental Table 1.

### MALDI MS imaging.

MALDI MS imaging of sulfatide and major phospholipids was carried out as previously described (80–82). Briefly, fresh frozen brains from C57BL/6 J WT and *db/db* mice were cryosectioned sagittally at a width of 10 μm using a Leica CM1900 cryostat (Leica Biosystems) at –12°C (chamber temperature). Tissue cryosections were then transferred to a polished stainless steel plate (Bruker Daltonics) and were desiccated in vacuum at room temperature for 30–60 minutes. A matrix solution, 1 mg/mL 9-aminoacridine in 90% methanol or 1 mg/mL N-(1-naphthyl) ethylenediamine dihydrochloride in 60% methanol, was sprayed using a manual airbrush equipped with a 0.2 mm nozzle (GSI Creos). The final deposition density was about 0.7 mg/cm^2^. Imaging data were acquired in the negative- or positive-ion mode using a Microglex MALDI-TOF mass spectrometer (Bruker Daltonics) equipped with a 337 nm N_2_ laser as the excitation source (150 μm laser rastering size). Imaging data for each pixel were summed up from 150 shots at a laser repetition rate of 30 Hz with intraspot rastering. Imaging data were analyzed using FlexImaging v3.0 (Bruker Daltonics). Ion images were generated with a bin width of ±0.2 Da. The normalization method was total ion count.

### Western blotting.

PNS and CNS tissues were homogenized in 1× NP-40. Western blotting was performed using NP-40 supernatants as previously described (81). Antibodies used in this study are detailed in Supplemental Table 2.

### Electron microscopy.

Mice were deeply anesthetized and transcardially perfused and postfixed for 2 weeks as previously described (83, 84). Sciatic nerves and spinal cords were harvested and processed for standard EM analysis as previously described (84, 85). Sciatic nerve and cervical spinal cord myelin thickness were calculated for WT and *db/db* mice. A minimum of 6 high-magnification light micrographs (1 μm thick) per animal were taken at original magnification, ×1200. For this analysis, only axons with diameters greater than 0.5 μm were counted as previously described (86). In addition, the electron micrographs were used to determine the g-ratio of the myelinated axons, calculated as the diameter of the axon divided by the diameter of the entire myelinated fiber.

### Electrophysiology.

Sciatic nerve conduction analyses were performed in vivo as previously described (87) on 2- and 4-month-old (8–9 weeks and 14–16 weeks, respectively) WT and *db/db* male mice (*n* = 5–6/genotype). Mice were anesthetized with urethane (1.5 mg/kg) IP. A ring recording electrode was placed over the gastrocnemius muscle with the reference electrode over its tendon, a stimulating cathode was placed 10–15 mm proximal to the recording electrode in the medial gluteal region to obtain proximal responses, and an anode electrode was placed subcutaneously in the midline over the sacrum. A monopolar disposable needle electrode was inserted under the skin of the mouse tail and used as a ground electrode. The sciatic nerve action potential was generated using a 0.1 ms biphasic pulse delivered every 20 seconds. Supramaximal response was gradually generated, and the current step was increased incrementally every 100 μA until the maximum amplitude of the action potential was reached (I/O curve). The CMAP amplitude was taken from peak to peak of the biphasic response.

### High-resolution respirometry.

HRR was conducted using 2 Oxygraph-2K (Oroboros Inc.) machines at 37°C in fresh spinal cord tissue homogenates and whole permeabilized sciatic nerves from 2-month-old WT and *db/db* mice. Whole spinal cord was homogenized with approximately 15 strokes using a Kontes glass homogenizer in 10% w/v ice-cold MiR06-Creatine (MiR05 + catalase + creatine) as previously described for brain tissue (88). Whole left and right sciatic nerves were dissected and permeabilized as previously described (23) with minor modifications. Briefly, sciatic nerves were kept at 4°C during the whole permeabilization process, which included equilibrating in MiR06 (1 minute), digesting with collagenase (1.5 mg/mL, 20 minutes), washing in MiR06 (10 minutes), and desheathing and defluffing using a pair of fine forceps. Spinal cord (2 mg) homogenates and whole permeabilized sciatic nerves from each genotype were loaded into separate 2 mL chambers and run simultaneously. Experiments were carried out when oxygen concentration in each well was saturated under supra-atmospheric conditions (~400 nM/mL O_2_) to ensure the reaction was not rate limited by oxygen. We followed the SUIT protocol as described previously (89), with minor modifications. Briefly, we added the following reagents as a single injection every ~5–10 minutes in the following order (final concentration): malate (2 mM), octanoylcarnitine (0.2 mM; FAO substrate), ADP (1.25 mM), glutamate (10 mM; CI substrate), 20 μL succinate (10 mM; CI+CII-linked respiration), ADP (1.25 mM; saturating concentration), 5 μL of cytochrome c (10 μM; to test the integrity of the outer mitochondrial membrane), FCCP (0.5 μM; uncoupler), 2 μL rotenone (1 μM; CI inhibitor), freshly prepared malonic acid (5 mM; CII inhibitor), and 1 μL antimycin A (2.5 μM; CIII inhibitor). The investigators performing the HRR were blinded to the sample group allocation during the experiment and analysis of the experimental outcome. Data were normalized to spinal cord mass or nerve bundle unit using Datlab software (Oroboros Inc.).

### Statistics.

Data sets were compared between genotypes, dietary regimens, or treatments by Student’s *t* tests (unpaired 2 tailed) or 2-way ANOVAs followed by Holm-Šidák multiple-comparisons tests (adjusted *P* values displayed) using GraphPad Prism software. See figure legends for specific *n* numbers and statistical tests used for each data set presented. Significance was set at the levels of *P* < 0.05 (*), < 0.01 (**), and < 0.001 (***) for all statistical comparisons. In addition, a *P* = 0.05–0.1 was considered a trend, and when appropriate the specific *P* value was specified. Lipidomics data, expressed as nmol of a given lipid class or molecular species per mg of total protein content, were either displayed as dot plots (data represent the mean ± SEM) for total class contents or transformed (log transformation) or displayed as gray scale heatmaps for molecular species within a given class.

### Study approval.

Protocols for all described animal experiments were approved by the UT Health SA Institutional Animal Care and Use Committee.

## Author contributions

JPP and JC share first coauthorship as they contributed similarly to data generation. JPP, DEK, NM, JLD, and XH designed and supervised research studies; JPP, JC, CW, JW, CQ, EB, XW, and JAW were responsible for conducting experiments, acquiring data, and analyzing data; JPP and XH wrote the first draft; and all authors reviewed and edited the draft.

## Supplementary Material

supplemental data

## Version 1 11/05/2020

Electronic publication
