# Supplementary material for: Early disruption of nerve mitochondrial and myelin lipid homeostasis in obesity-induced diabetes
Source: JCI Insight. 2020 Nov 5;5(21):e137286. doi: 10.1172/jci.insight.137286 (PMC7710310; doi:10.1172/jci.insight.137286)

SUPPLEMENTAL MATERIAL

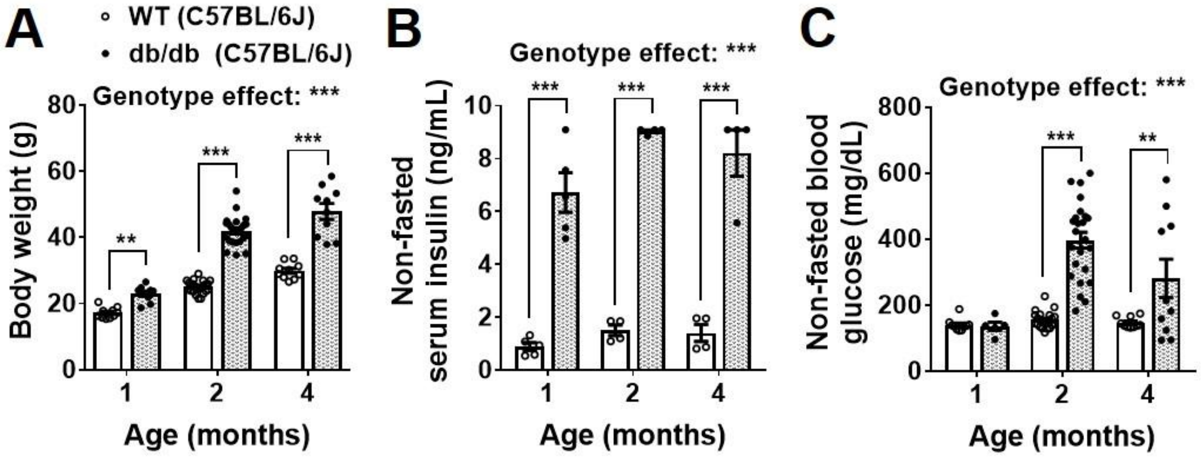

Supplemental Figure 1. Body weight, insulin, and glucose levels in *db/db* mice compared to WT controls. Body weight (**A**), non-fasted serum insulin (**B**), and non-fasted blood glucose (**C**) were measured for WT (open circles/bars) and *db/db* (filled circles/bars) mice at 1, 2, and 4 months of age. Graphs are presented as dot plots with bars, each dot represents a different animal, data represent mean ± SEM. n = 9-24 male mice/genotype/time point for panels A and C, n = 4-5 male mice/genotype/time point for panel B. Each parameter was compared between genotypes and time points using 2-way ANOVA and Sidak's multiple comparisons tests on GraphPad Prism 7. ** p <0.01, *** $p < 0.001$.

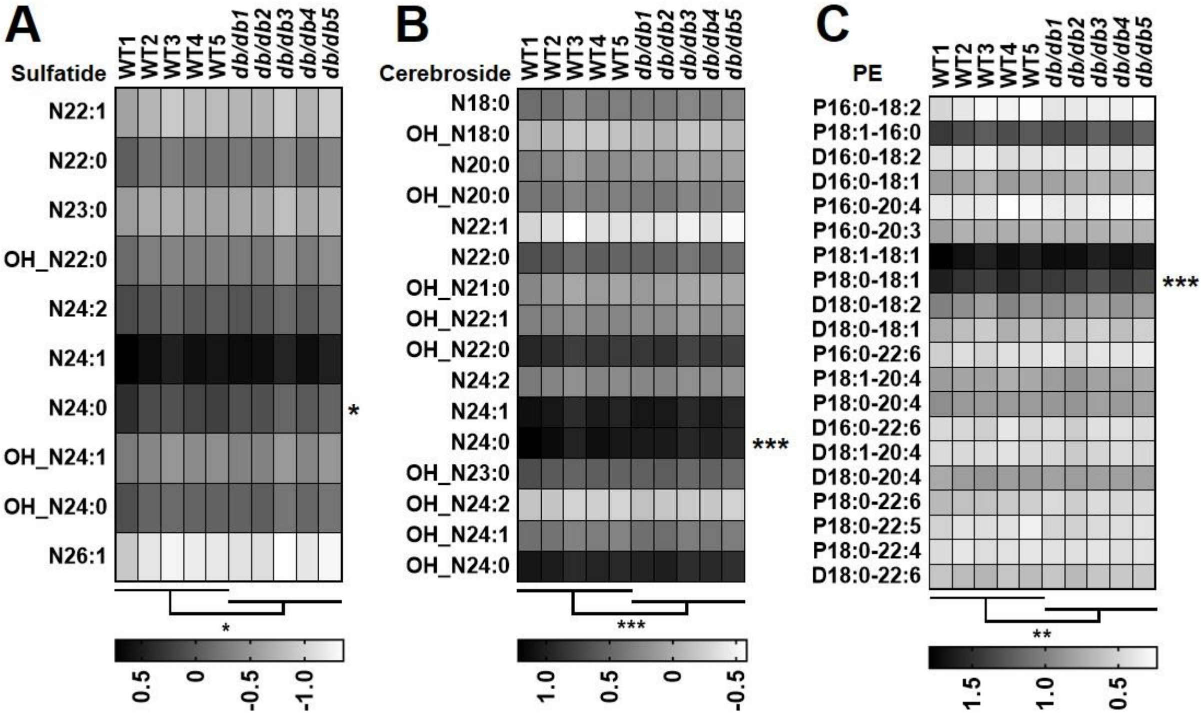

Supplemental Figure 2. Weak myelin lipid alterations at the molecular species level in the dorsal root ganglia of *db/db* mice at 2 months of age. Dorsal root ganglia (DRG) from 2-month-old WT and *db/db* male mice were dissected, flash frozen, homogenized, and lipid extracted. Sulfatide (**A**), cerebroside (**B**), and phosphatidylethanolamine (PE) (**C**) levels were assessed by multi-dimensional mass spectrometry-based shotgun lipidomics. Lipid masses expressed as nmol/mg of total protein were log transformed and displayed as gray scale heat maps. Although a specific mass peak may represent more than one molecular species, for simplicity heatmaps display only one (most common) lipid species for each mass (row). Molecular species within each lipid class were compared between genotypes using 2-way ANOVA and Sidak's multiple comparisons tests using non-transformed data on GraphPad Prism 7. Low abundant lipid species that made < 1% of the total class were excluded. N, amide-linked; P, plasmalogen or alkenyl-acyl-linked; D, diacyl-linked. $n = 5$ mice/genotype for all panels. * $p < 0.05$, ** $p < 0.01$, *** $p < 0.001$.

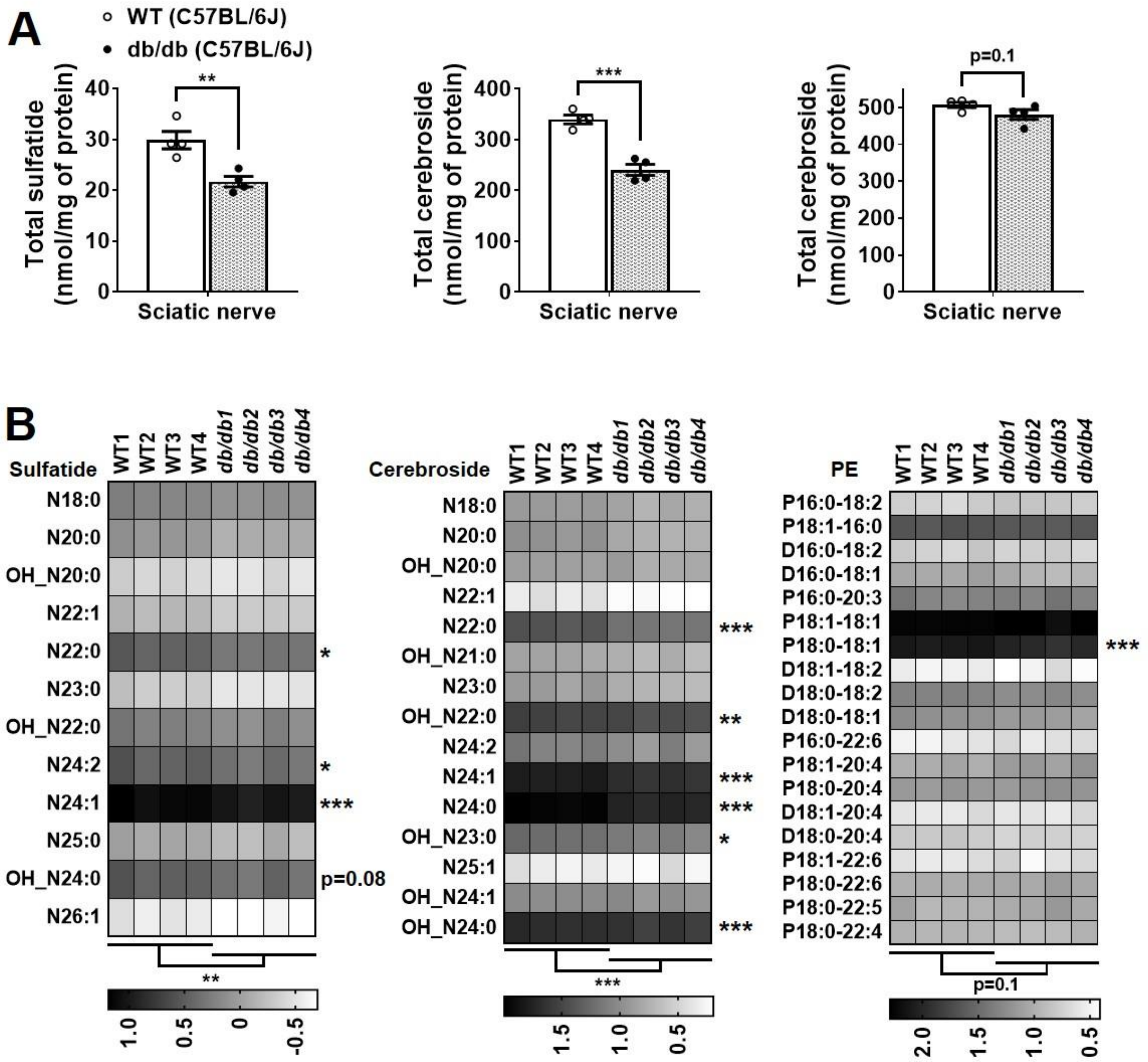

Supplemental Figure 3. Sustained global myelin lipid deficiency in the nerves obese diabetic mice. Sciatic nerve tissue from 4-month-old WT and *db/db* male mice were dissected, flash frozen, homogenized, and lipid extracted. (**A**) Total levels of sulfatide, cerebroside, and phosphatidylethanolamine (PE), assessed by multi-dimensional mass spectrometry-based shotgun lipidomics. Graphs are presented as dot plots with bars, each dot represents a different animal, data represent mean ± SEM. Comparisons between genotypes for each lipid class (total content) were performed separately using unpaired two-tailed Student's *t* tests. (**B**) Molecular species masses for each lipid class, expressed as nmol/mg of total protein, were log transformed and displayed as gray scale heat maps. Although a specific mass peak may represent more than one molecular species, for simplicity only one (most common) lipid species for each mass (row) is listed. Molecular

species within each lipid class were compared between genotypes using 2-way ANOVA and Sidak's multiple comparisons tests using non-transformed data on GraphPad Prism 7. Low abundant lipid species that made < 1% of the total class were excluded. N, amide-linked; P, plasmalogen or alkenyl-acyl-linked; D, diacyl-linked. $n = 4$ mice/genotype for all panels. * $p < 0.05$, ** $p < 0.01$, *** $p < 0.001$.

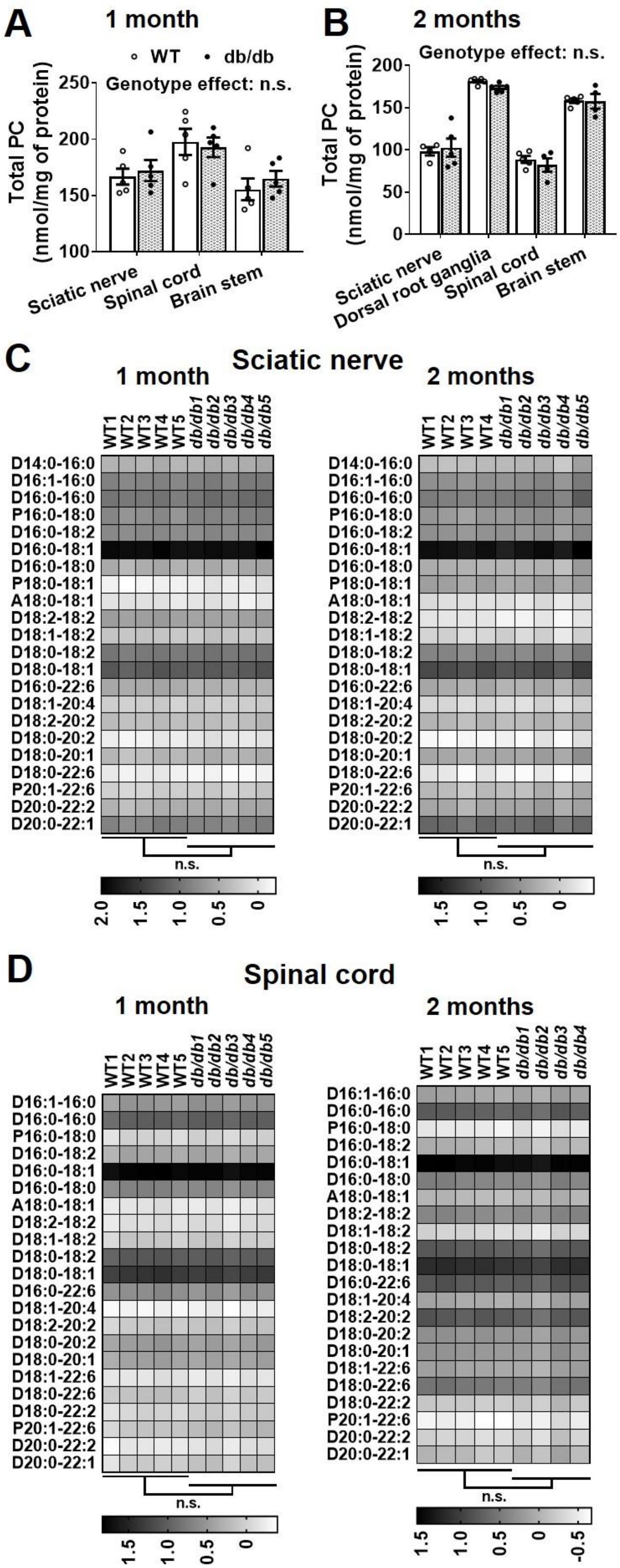

A
1 month
WT
db/db
Genotype effect: n.s.
Total PC
(nmol/mg of protein)
250
200
150
100
Sciatic nerve
Spinal cord
Brain stem
B
2 months
Genotype effect: n.s.
Total PC
(nmol/mg of protein)
200
150
100
50
0
Sciatic nerve
Dorsal root ganglia
Spinal cord
Brain stem
C
Sciatic nerve
1 month
WT1
WT2
WT3
WT4
WT5
db/db1
db/db2
db/db3
db/db4
db/db5
D14:0-16:0
D16:1-16:0
D16:0-16:0
P16:0-18:0
D16:0-18:2
D16:0-18:1
D16:0-18:0
P18:0-18:1
A18:0-18:1
D18:2-18:2
D18:1-18:2
D18:0-18:2
D18:0-18:1
D16:0-22:6
D18:1-20:4
D18:2-20:2
D18:0-20:2
D18:0-20:1
D18:0-22:6
P20:1-22:6
D20:0-22:2
D20:0-22:1
n.s.
2.0
1.5
1.0
0.5
0
2 months
WT1
WT2
WT3
WT4
db/db1
db/db2
db/db3
db/db4
db/db5
D14:0-16:0
D16:1-16:0
D16:0-16:0
P16:0-18:0
D16:0-18:2
D16:0-18:1
D16:0-18:0
P18:0-18:1
A18:0-18:1
D18:2-18:2
D18:1-18:2
D18:0-18:2
D18:0-18:1
D16:0-22:6
D18:1-20:4
D18:2-20:2
D18:0-20:2
D18:0-20:1
D18:0-22:6
P20:1-22:6
D20:0-22:2
D20:0-22:1
n.s.
1.5
1.0
0.5
0
D
Spinal cord
1 month
WT1
WT2
WT3
WT4
WT5
db/db1
db/db2
db/db3
db/db4
db/db5
D16:1-16:0
D16:0-16:0
P16:0-18:0
D16:0-18:2
D16:0-18:1
D16:0-18:0
A18:0-18:1
D18:2-18:2
D18:1-18:2
D18:0-18:2
D18:0-18:1
D16:0-22:6
D18:1-20:4
D18:2-20:2
D18:0-20:2
D18:0-20:1
D18:1-22:6
D18:0-22:6
D18:0-22:2
P20:1-22:6
D20:0-22:2
D20:0-22:1
n.s.
1.5
1.0
0.5
0
2 months
WT1
WT2
WT3
WT4
WT5
db/db1
db/db2
db/db3
db/db4
D16:1-16:0
D16:0-16:0
P16:0-18:0
D16:0-18:2
D16:0-18:1
D16:0-18:0
A18:0-18:1
D18:2-18:2
D18:1-18:2
D18:0-18:2
D18:0-18:1
D16:0-22:6
D18:1-20:4
D18:2-20:2
D18:0-20:2
D18:0-20:1
D18:1-22:6
D18:0-22:6
D18:0-22:2
P20:1-22:6
D20:0-22:2
D20:0-22:1
n.s.
1.5
1.0
0.5
0
-0.5

Supplemental Figure 4. Major cellular phospholipid homeostasis unaltered in *db/db* mice. Sciatic nerve, dorsal root ganglia, spinal cord, and/or brain stem tissue from for WT (open circles/bars) and *db/db* (filled circles/bars) male mice were dissected, flash frozen, homogenized, and lipid extracted. Total phosphatidylcholine (PC) levels were assessed by multi-dimensional mass spectrometry-based shotgun lipidomics for 1- (**A**) and 2- (**B**) month-old mice. Total levels of each lipid class are displayed as dot plots with bars, data represent mean ± SEM (**A** and **C**). Total levels of each lipid class were compared between genotypes for every tissue examined at each time point using 2-way ANOVA and Sidak's multiple comparisons tests. PC molecular species masses, expressed as nmol/mg of total protein, were log transformed and displayed as gray scale heat maps for sciatic nerve (**C**) and spinal cord tissue (**D**). Although a specific mass peak may represent more than one molecular species, for simplicity only one (most common) lipid species for each mass (row) is listed. Molecular species within each lipid class were compared between genotypes using 2-way ANOVA and Sidak's multiple comparisons tests using non-transformed data on GraphPad Prism 7. Low abundant lipid species that made < 1% of the total class were excluded. N, amide-linked; P, plasmalogen or alkenyl-acyl-linked; D, diacyl-linked. n = 4-5 mice/genotype for all panels. n.s.: not significant.

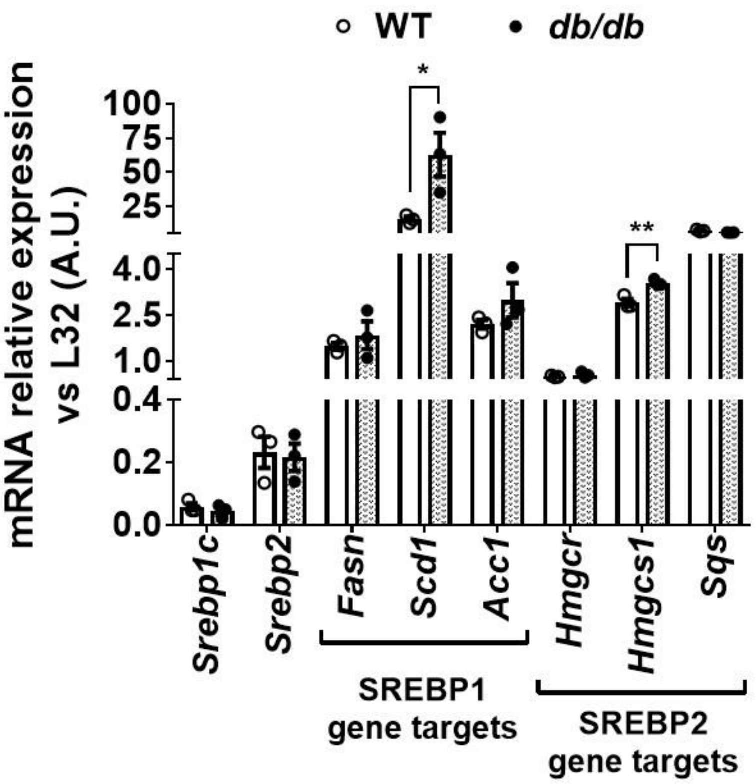

Supplemental Figure 5. Lipid biosynthesis-related genes are not downregulated in *db/db* mice. Total RNA was isolated after homogenizing sciatic nerves from 2-month-old WT (open circles/bars) and *db/db* (filled circles/bars) male mice, mRNA was reverse transcribed, and qRT-PCR was performed using SsoFast™ EvaGreen® Supermix (Bio-Rad). L32 expression was used to normalize samples and obtain relative expression. Comparisons between genotypes were performed separately using an unpaired two-tailed Student's *t* test for each gene on GraphPad Prism 7. Graphs are presented as dot plots with bars, each dot represents data from a different animal (run in duplicate and averaged), data represent mean ± SEM. $n = 3$ males/genotype. * $p < 0.05$, ** $p < 0.01$. *Srebp1/2*, sterol regulatory element binding protein 1/2; *Fasn*, fatty acid synthase; *Scd1*, stearoyl-CoA desaturase-1; *Acc1*, acetyl-CoA carboxylase 1; *Hmgcr,* 3-hydroxy-3-meScthyl-glutaryl-coenzyme A (HMG-CoA) reductase; *Hmgcs1*, HMG-CoA synthase 1; *Sqs*, squalene synthase. *Srebp*1a levels were below detection limits (not shown).

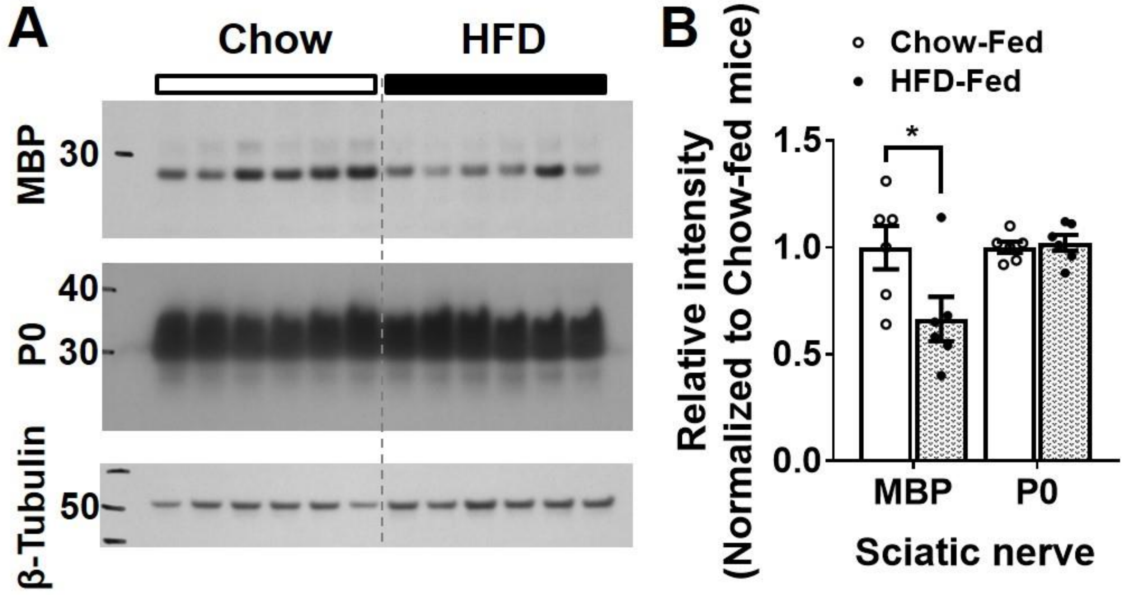

Supplemental Figure 6. Alterations in specific myelin-specific proteins in diet induced obese pre-diabetic mice. (**A**) Representative Western blots from sciatic nerve NP40 homogenates (supernatants) of chow- (open circles/bars) and high fat diet (HFD)-fed (filled circles/bars) mice using antibodies against MBP and P0 (MPZ). Relative intensities (normalized to Chow-fed mice) were quantified using ImageJ software. β-Tubulin is shown as an example of a loading control. (**B**) Graphs are presented as dot plots with bars, each dot represents a different animal, data represent mean ± SEM. $n = 6$ males/dietary regimen. Comparisons between genotypes for each protein were performed separately using unpaired two-tailed Student's *t* tests. * $p < 0.05$. Full/unedited versions of the gels provided in the Supplemental Material.

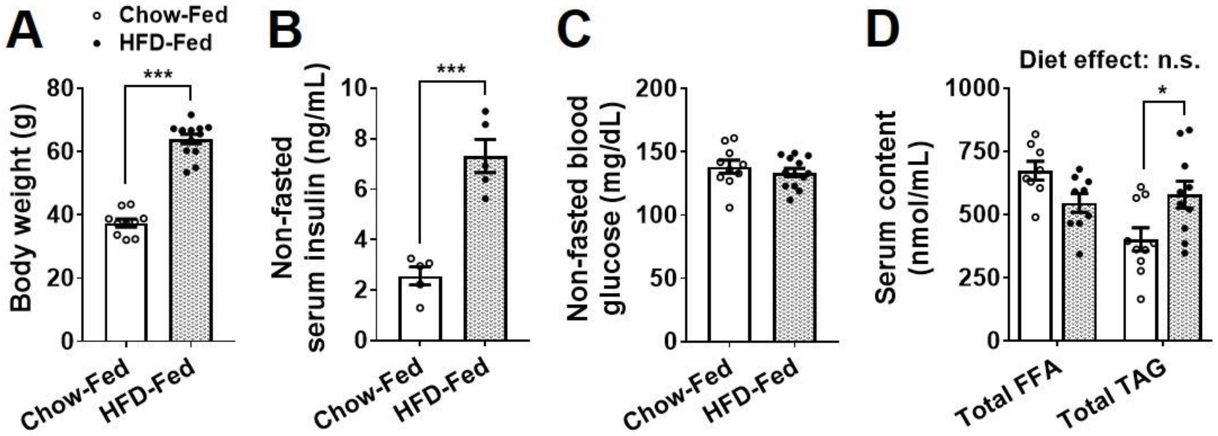

Supplemental Figure 7. Body weight and circulating levels of insulin, glucose, free fatty acids, and triglycerides in chow-fed and HFD-fed mice. Body weight (**A**) and non-fasted levels of serum insulin (**B**), blood glucose (**C**), serum free fatty acids (FFA, **D**) and triglycerides (TAG, **D**) for chow-fed (open circles/bars) and HFD-fed (filled circles/bars) mice were measured at the endpoint of the experiment. Comparisons between dietary regimens for each measurement and time point were performed separately using unpaired two-tailed Student's *t* tests. Serum free fatty acids (FFA) and triacylglycerols (TAG) were assessed by shotgun lipidomics. Lipid classes were compared between groups using 2-way ANOVA and Sidak's multiple comparisons tests using non-transformed data on GraphPad Prism 7. Graphs are presented as dot plots with bars, each dot represents a different animal, data represent mean ± SEM. n = 10-13 mice/dietary regimen for all panels except for B (n = 5 males/dietary regimen). * p <0.05, *** p < 0.001. n.s.: not significant.

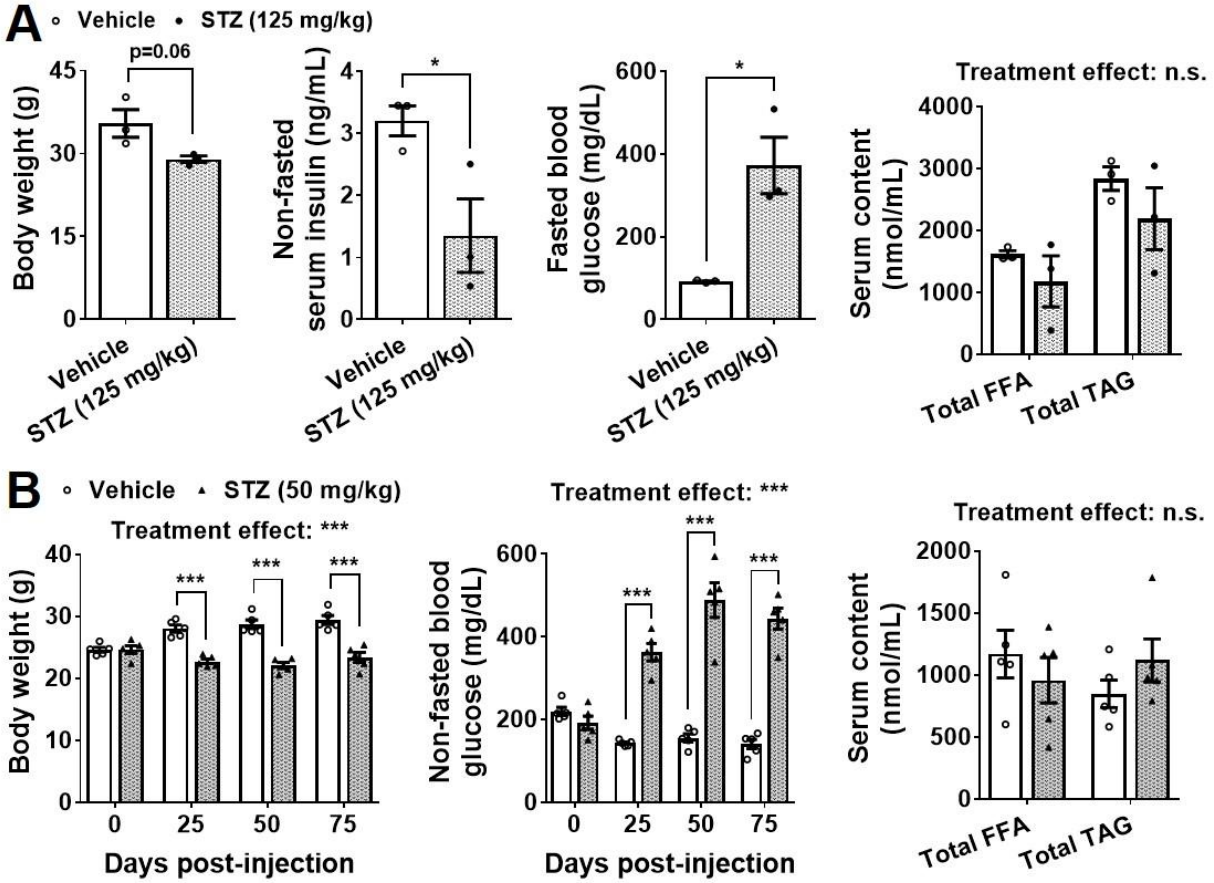

Supplemental Figure 8. Body weight, insulin, and glucose levels in vehicle and STZ-treated mice. (A) Body weight, non-fasted levels of serum insulin, and blood glucose for high-dose (125 mg/kg) short-term (10 days) STZ-treated mice (open circles/bars) and vehicle-treated control mice (filled circles/bars). Comparisons between treatments (vehicle vs STZ) for each of the above mentioned measurements were performed separately using unpaired two-tailed Student's *t* tests. (**B**) Body weight and non-fasted levels of blood glucose were measured every 25 days for low-dose (50 mg/kg x 5) long-term (2.5 months) STZ-treated (open circles/bars) and control (filled triangles/bars) mice. Serum free fatty acids (FFA) and triacylglycerols (TAG) were assessed by shotgun lipidomics for both treatments (**A** and **B**). Graphs are presented as dot plots with bars, each dot represents a different animal, data represent mean ± SEM. $n = 3$ males/treatment (**A**); $n = 5$ males/treatment for panel (**B**). Multiple time points, as well as lipid classes were compared between groups using 2-way ANOVA and Sidak's multiple comparisons tests on GraphPad Prism 7. * p <0.05, *** $p < 0.001$. n.s.: not significant.

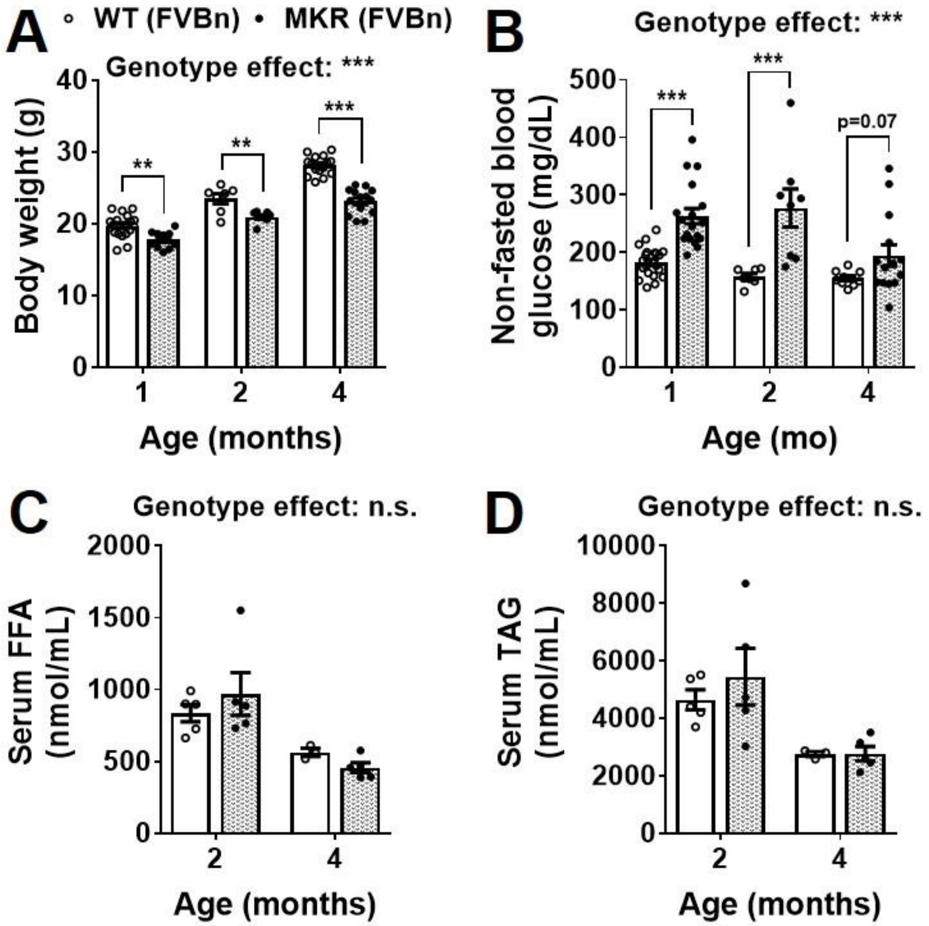

Supplemental Figure 9. Body weight, insulin, and glucose levels in MKR mice compared to WT controls. Body weight (**A**), non-fasted levels of blood glucose (**B**), serum free fatty acids (FFA, **C**) and triglycerides (TAG, **D**) for WT (open circles/bars) and MKR (filled circles/bars) male mice (both on a FVBn genetic background) were measured at the endpoint of the experiment. Graphs are presented as dot plots with bars, each dot represents a different animal, data represent mean ± SEM. ** p <0.01, *** p < 0.001, n.s.: not significant. n = 8-22 mice/genotype/time point (**A**-**B**); 3-5 mice/genotype/time point for panels (**C**-**D**). Genotypes were compared for each parameter at different time points using 2-way ANOVA and Sidak's multiple comparisons tests on GraphPad Prism 7.

Supplemental Table 1. Primer sequences used for gene expression studies.

**Name**	**Sequence (5' → 3')**
mSREBP-1A_cDNA_F	CACAGCGGTTTCGAACG
mSREBP-1C_cDNA_F	TGGATTGCACATTTGAAGACAT
mSREBP-1_cDNA_R	GCCAGAGAAGCAGAAGAG
mSREBP-2_cDNA_F	ATGGAGACCCTCACGGA
mSREBP-2_cDNA_R	TGCTGTTGTTGCCACTG
mFASN(B&G)-F	GCTGCGGAAACTTCAGGAAAT
mFASN(B&G)-R	AGAGACGTGTCACTCCTGGACTT
mSCD-1 (F)	CCGGAGACCCCTTAGATCGA
mSCD-1 (R)	TAGCCTGTAAAAGATTTCTGCAAACC
mACC1-F	TGACAGACTGATCGCAGAGAAAG
mACC1-R	TGGAGAGCCCCACACACA
mHMGCR-F	CTTGTGGAATGCCTTGTGATTG
mHMGCR-R	AGCCGAAGCAGCACATGAT
mSQS-F	CCAACTCAATGGGTCTGTTCCT
mSQS-R	TGGCTTAGCAAAGTCTTCCAACT
mL32-F	ACATTTGCCCTGAATGTGGT
mL32-R	ATCCTCTTGCCCTGATCCTT

Supplemental Table 2. Antibodies used for Western blotting.

Antibody Name	Host	Company	Catalog Number
Oxphos Cocktail	Mouse	Abcam	Ab110413
MPZ (P0)	Chicken	Novus	NB100-1607
PLP1	Rabbit	Sigma	HPA004128
MBP	Rabbit	Cell Signaling	#78896
CNP	Rabbit	Cell Signaling	#5664
UCH-L/PGP9.5	Rabbit	Novus Biologicals	NB110-58874
GAPDH	Mouse	Millipore	#MAB374
β-Tubulin	Rabbit	Cell Signaling	#2146

Full unedited gels for Figure 7A (Sciatic nerve, 1 month-old)

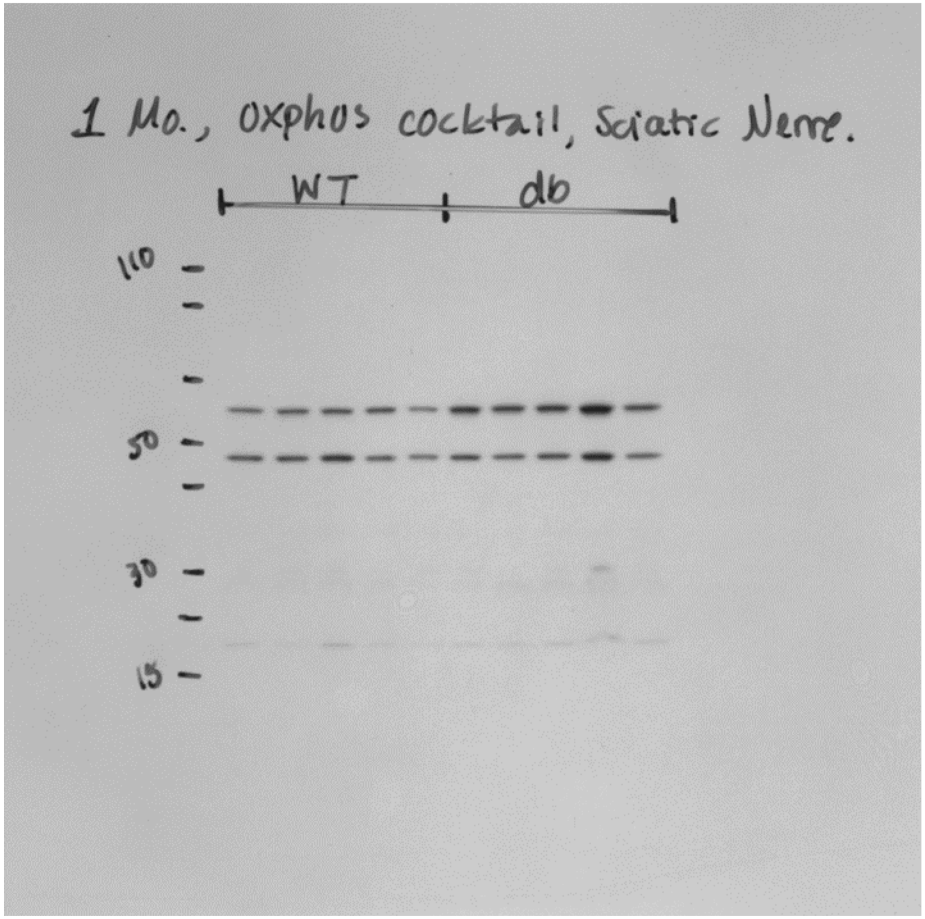

Low exposure

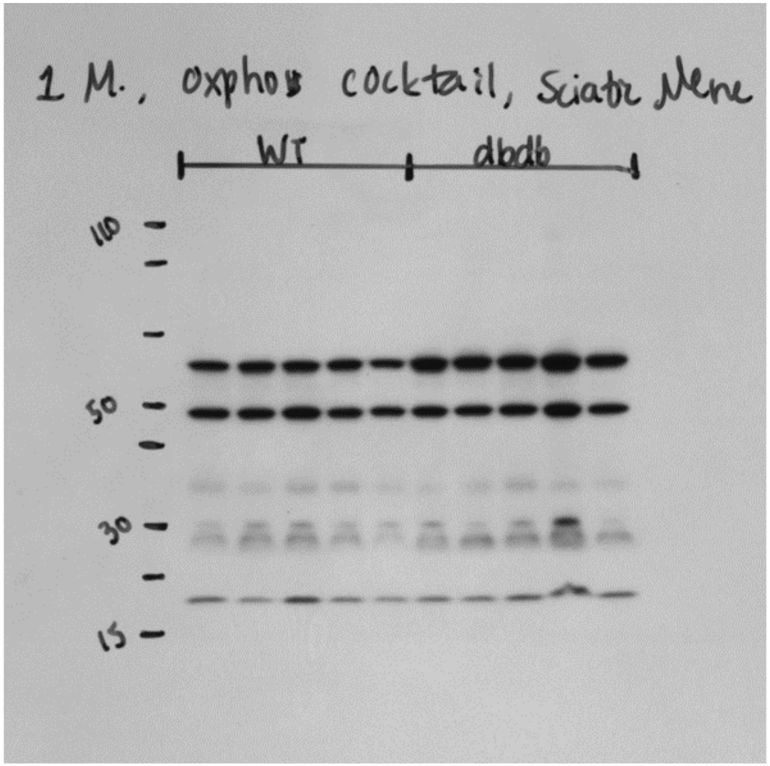

High exposure

Full unedited gels for Figure 7A (Sciatic nerve, 1-month-old)

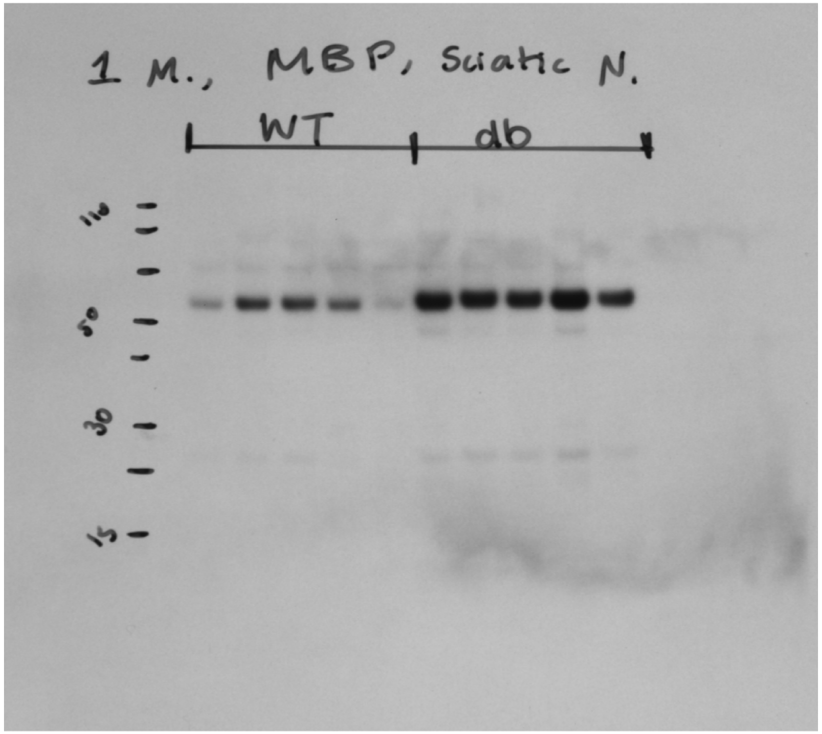

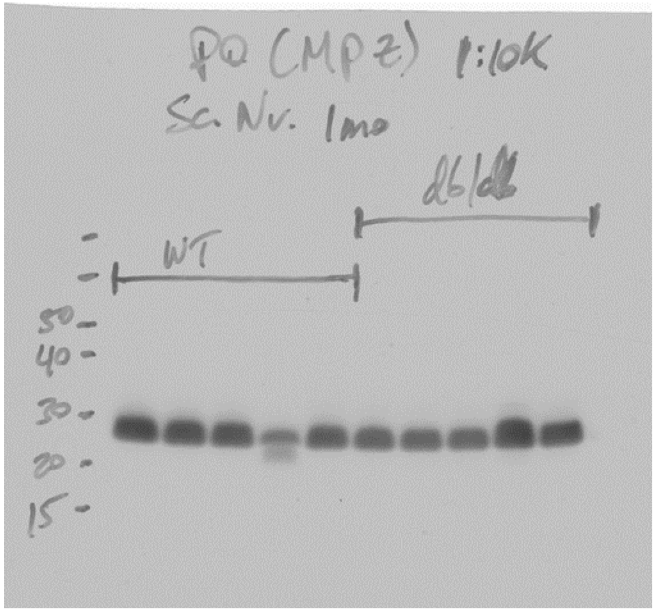

Full unedited gels for Figure 7A (Sciatic nerve, 1-month-old)

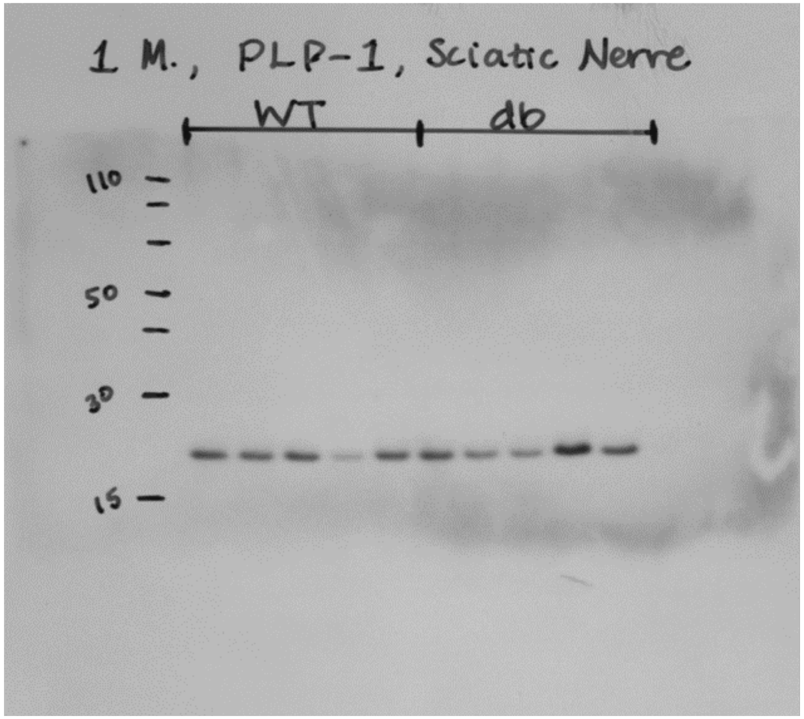

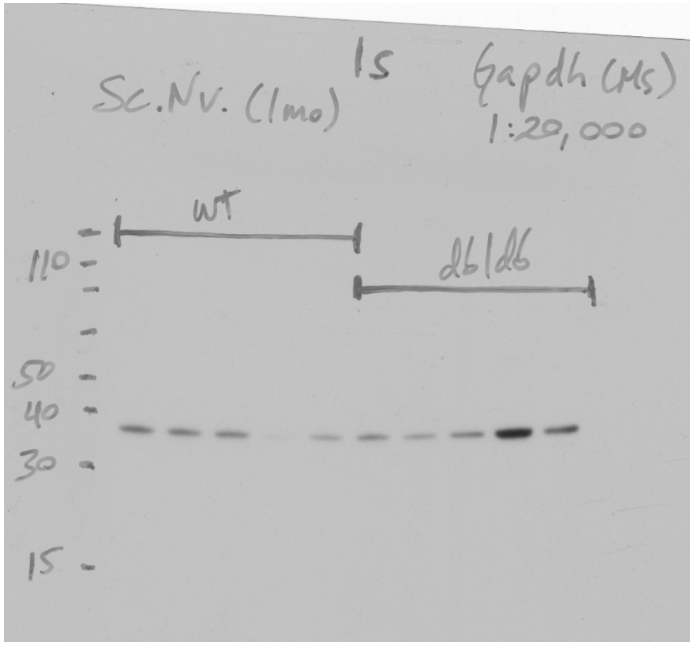

Full unedited gels for Figure 7B (Sciatic nerve, 2-month-old)

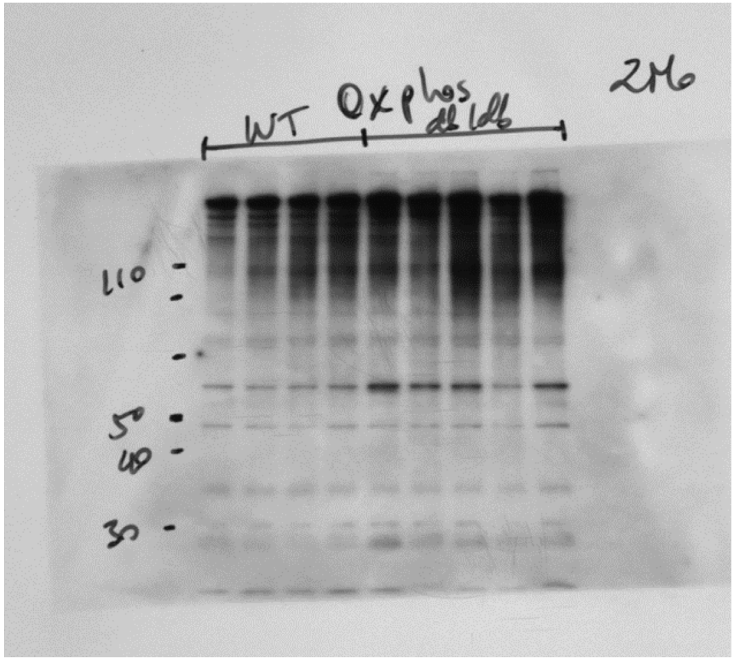

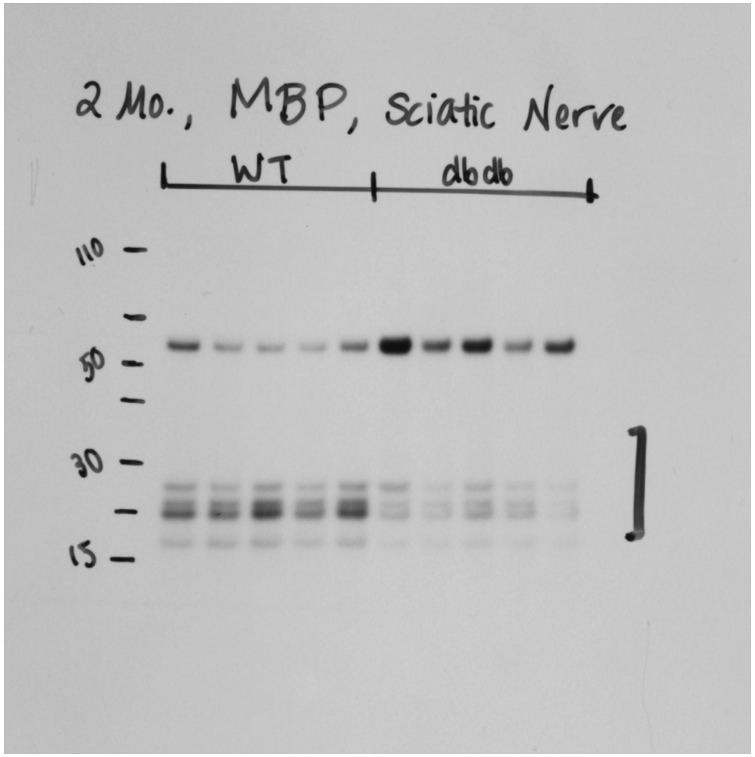

Note: blot was reblotted after using an antibody against phospho-neurofilament H (top bands)

Full unedited gels for Figure 7B (Sciatic nerve, 2-month-old)

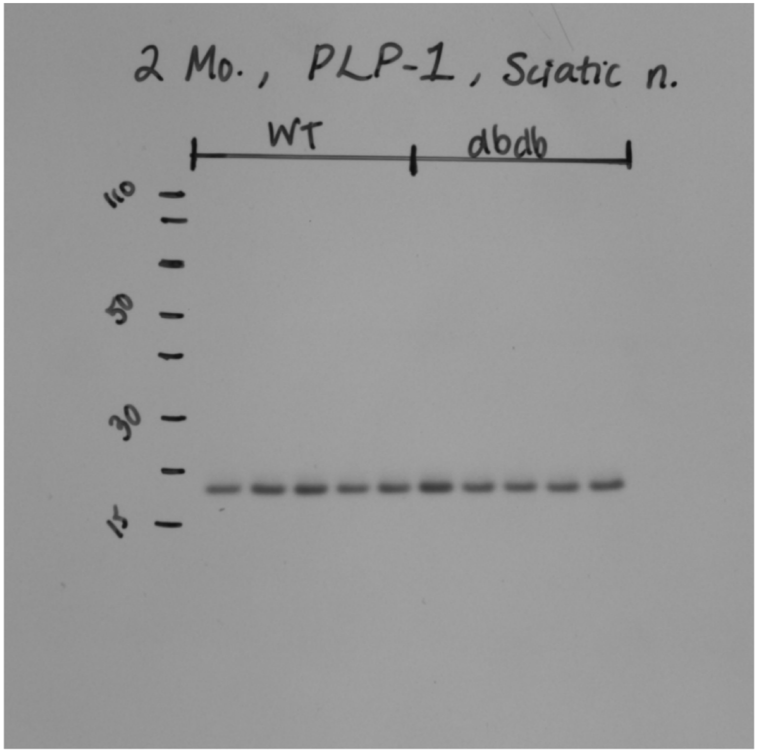

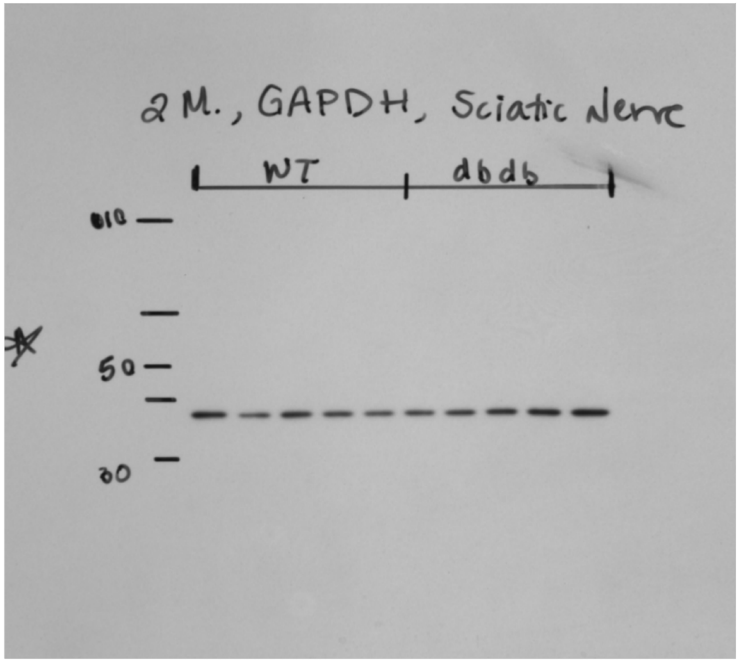

Full unedited gels for Figure 7C (Sciatic nerve, 4-month-old)

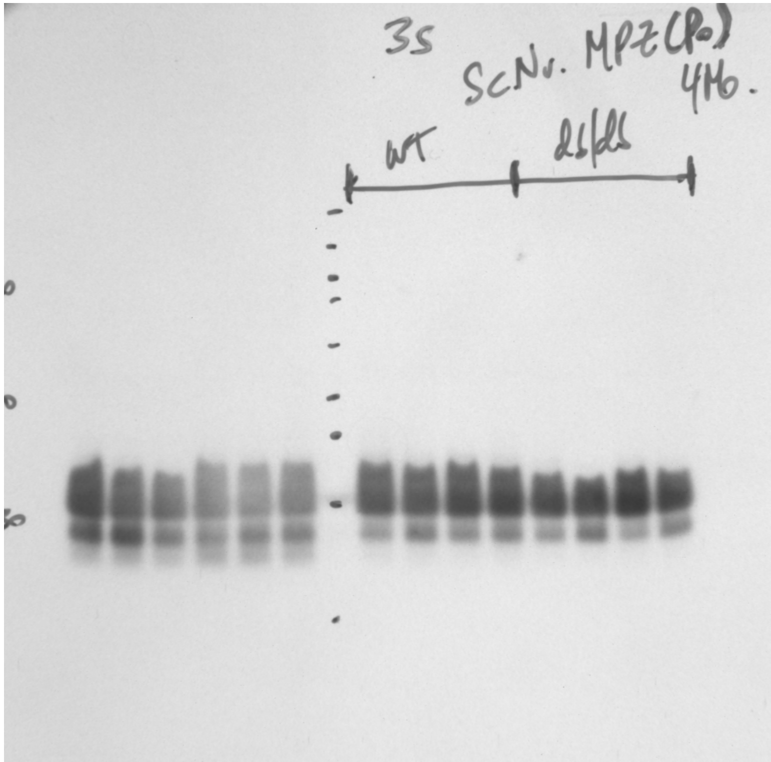

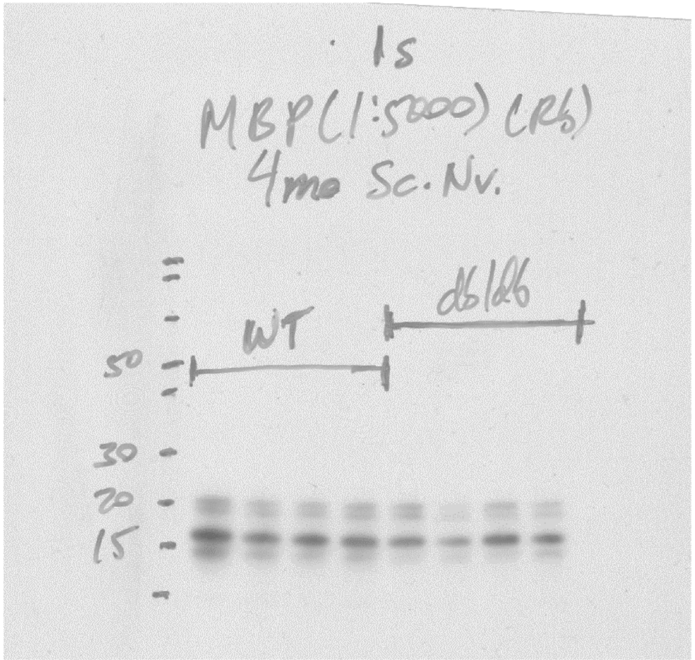

Full unedited gels for Figure 7C (Sciatic nerve, 4-month-old)

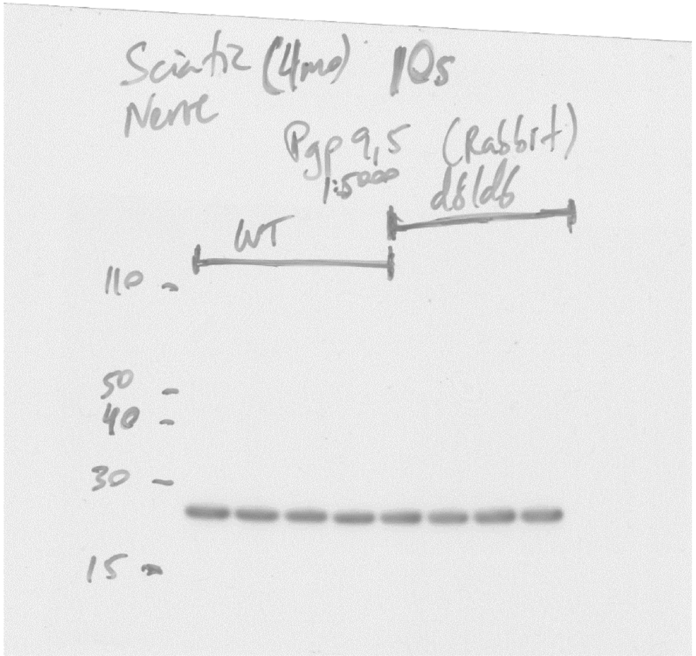

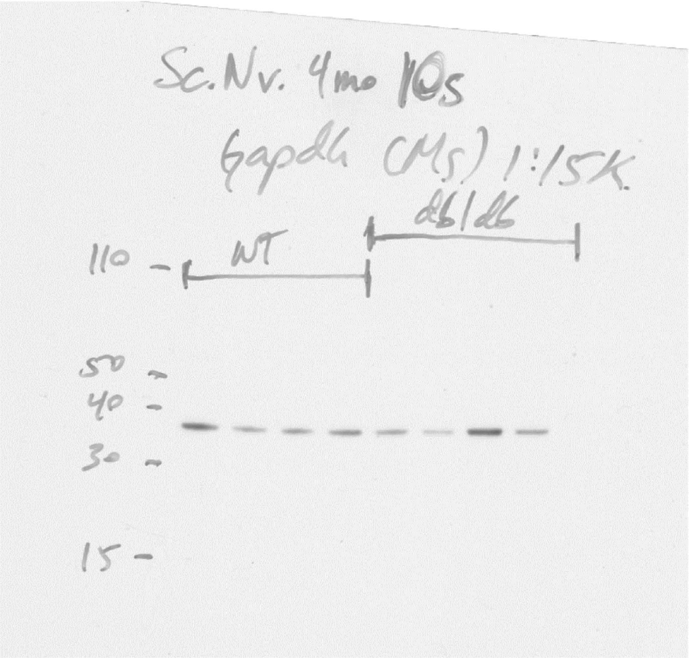

Full unedited gels for Figure 7D (Spinal cord, 1-month-old)

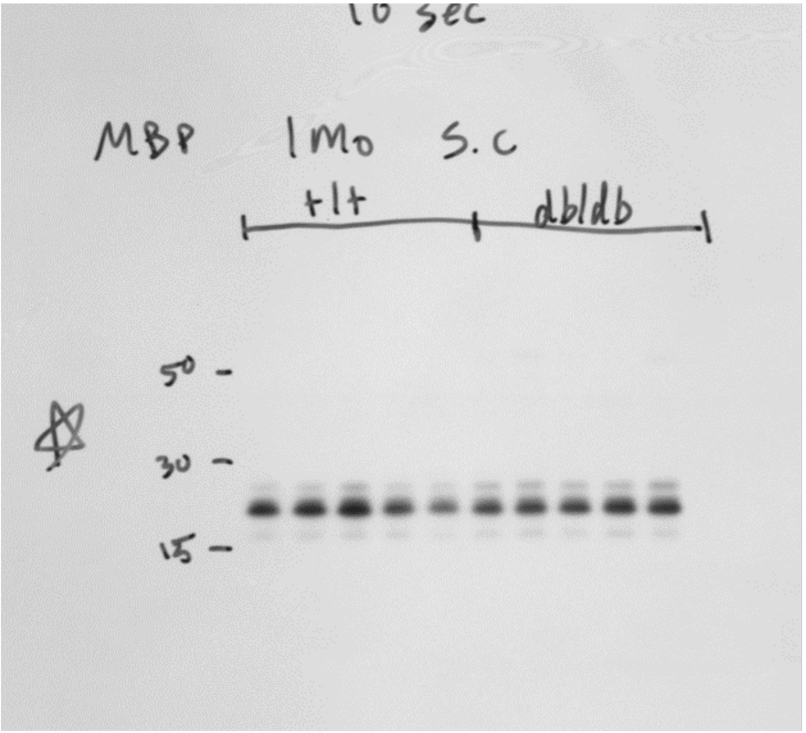

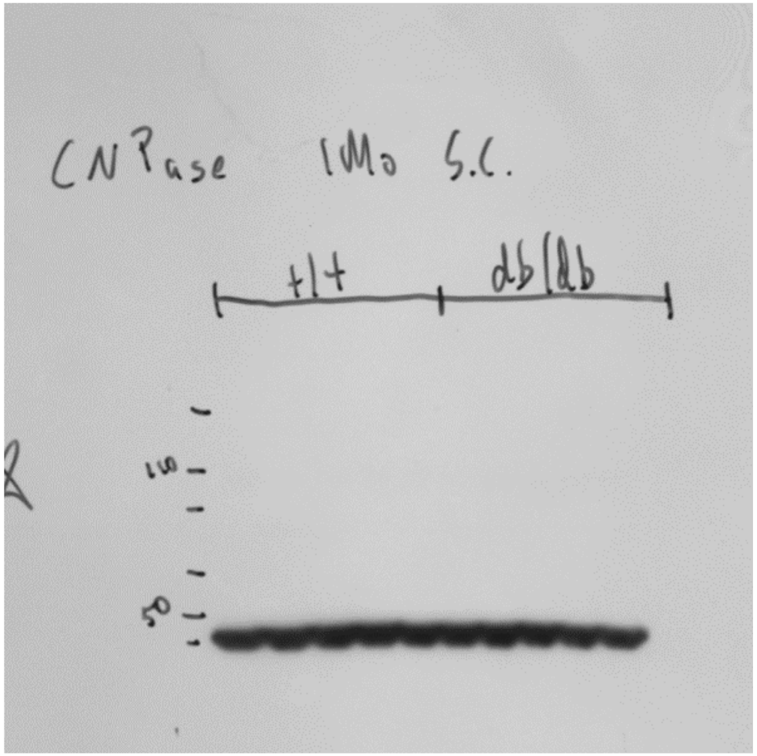

Full unedited gels for Figure 7D (Spinal cord, 1-month-old)

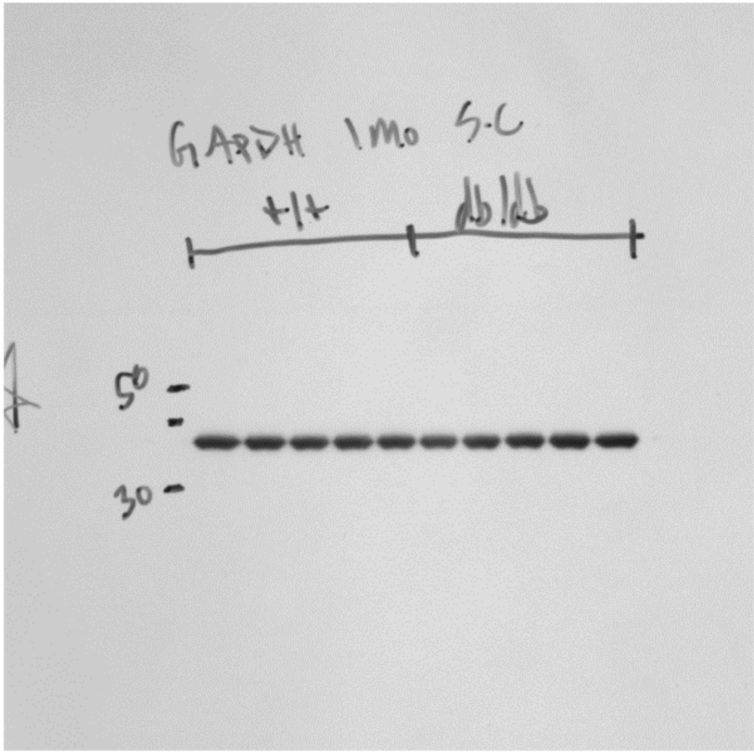

Full unedited gels for Figure 7E (Brain stem, 2-month-old)

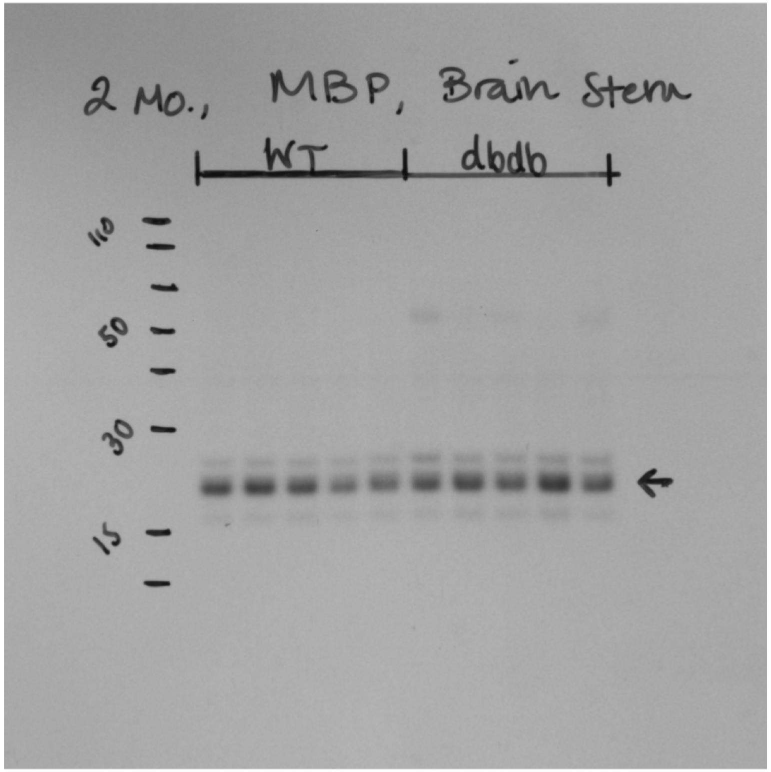

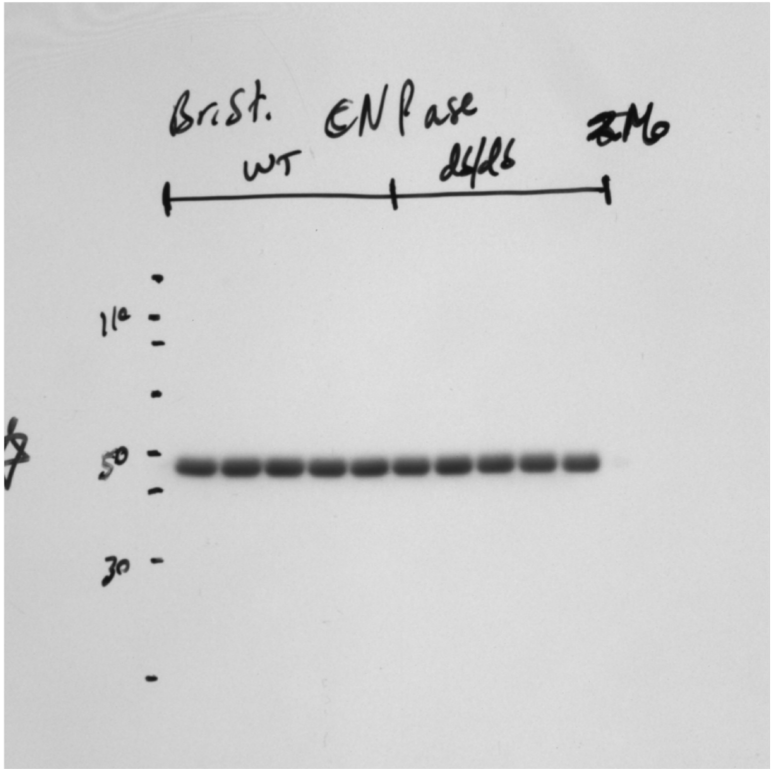

Full unedited gels for Figure 7B (Brain stem, 2-month-old)

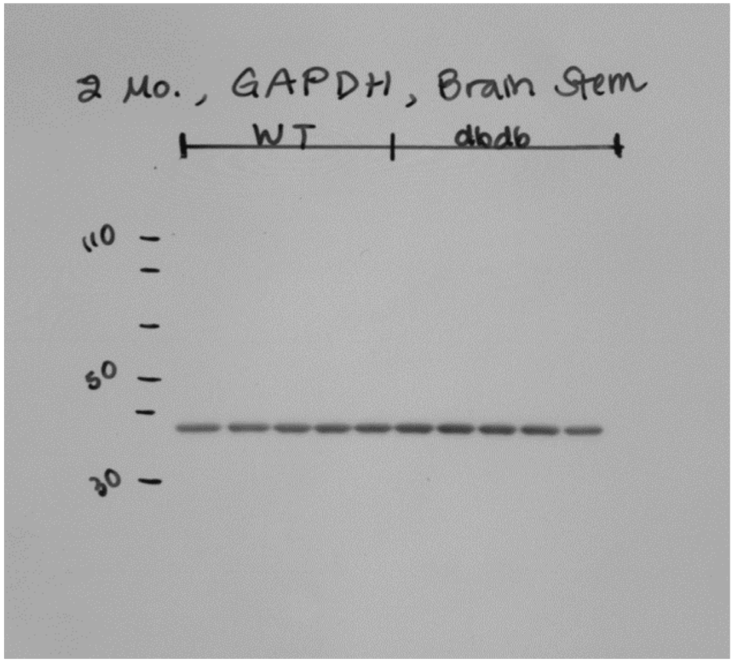

Full unedited gels for Figure 7F (Spinal cord, 4-month-old)

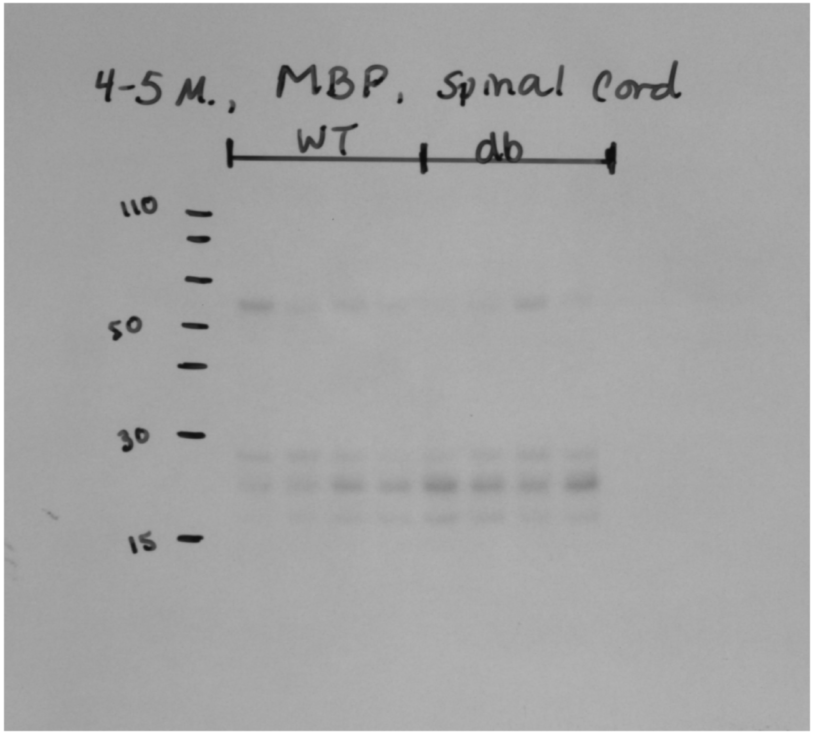

Full unedited gels for Figure 7F (Spinal cord, 4-month-old)

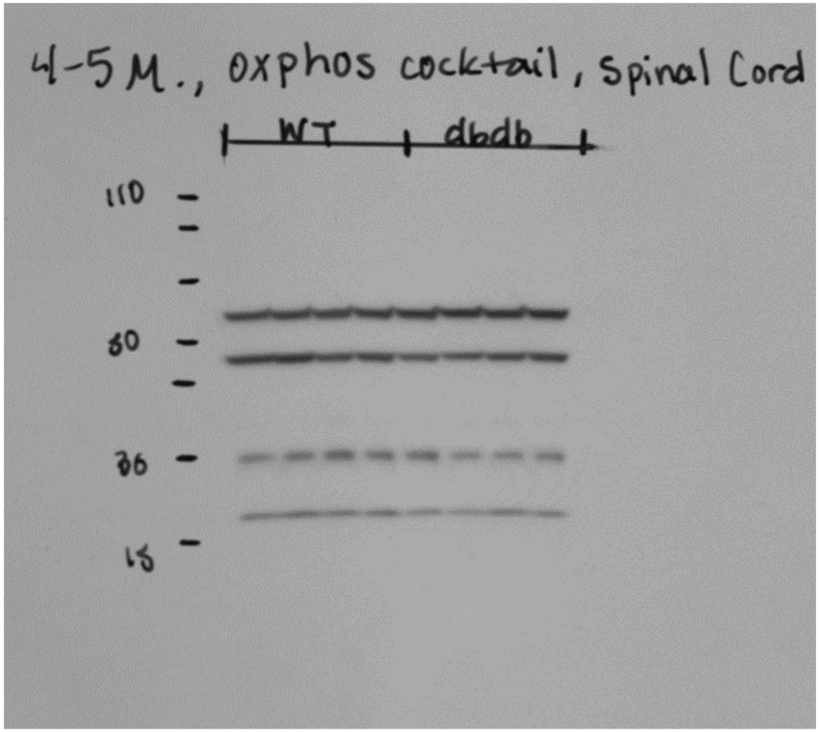

Low exposure

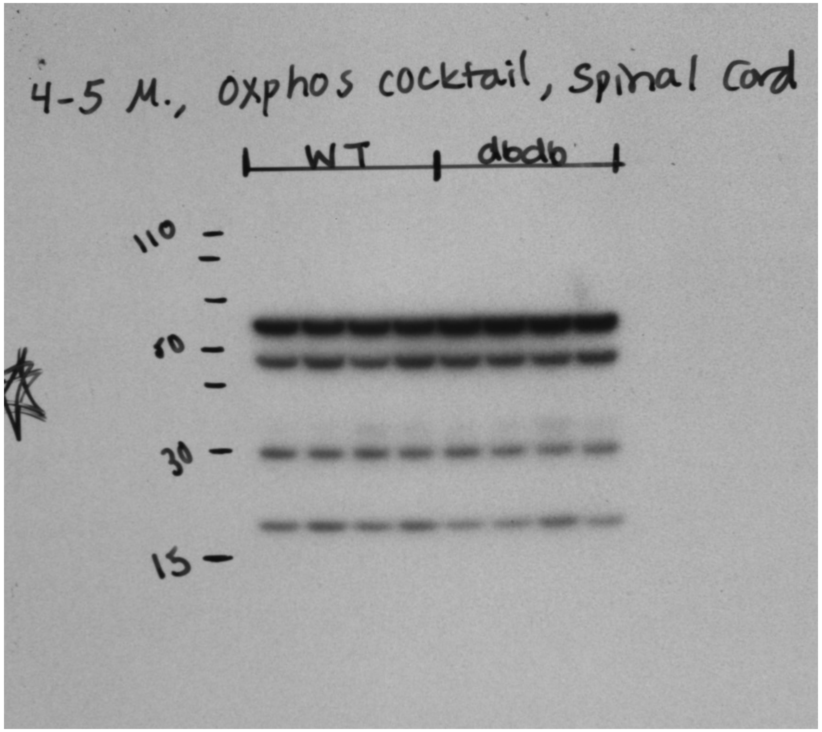

High exposure

Full unedited gels for Figure 7F (Spinal cord, 4-month-old)

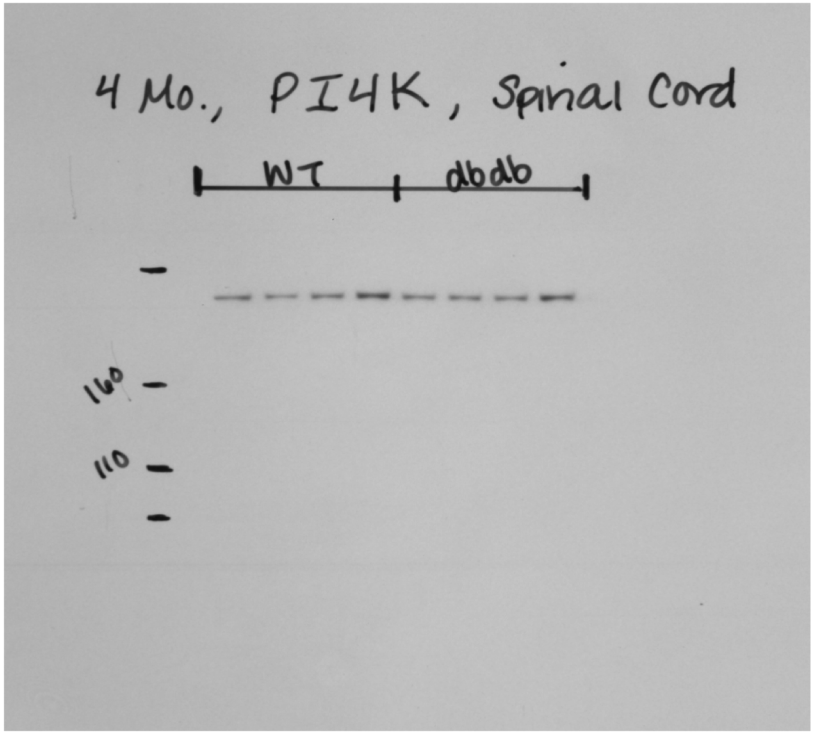

Note: Although GAPDH was not included as a loading control in this particular time point/region, we tested for additional proteins, like PI4K, that were not altered and can also serve as loading controls.

Full unedited gels for Supplemental Figure 6 (Chow vs HFD)

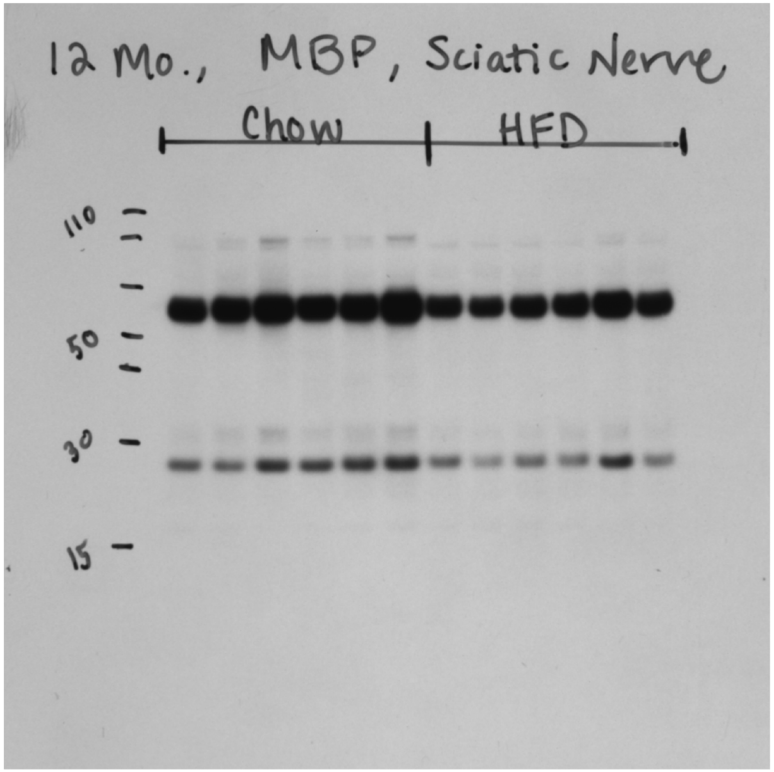

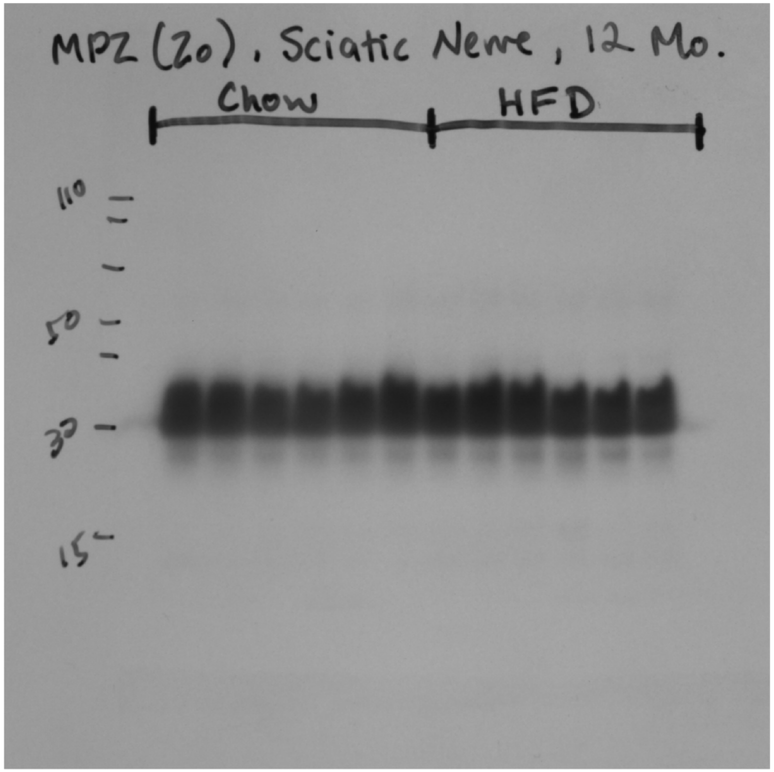

Full unedited gels for Supplemental Figure 6 (Sciatic nerve, Chow vs HFD)

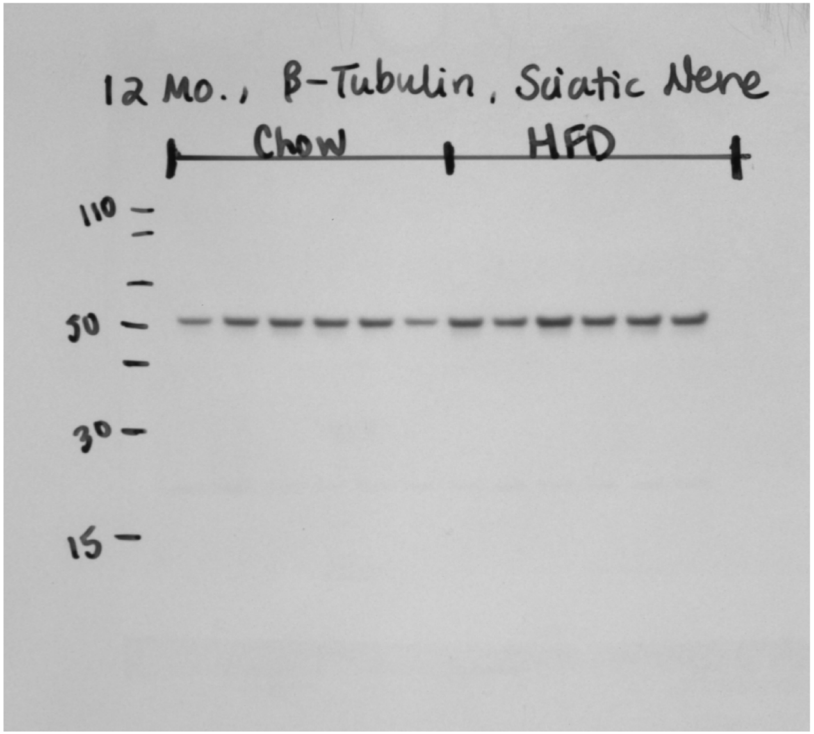

Supplement: supplemental data [file jciinsight-5-137286-s032.pdf]
